# Design, Synthesis and Insecticidal Activity of Novel Phenylurea Derivatives

**DOI:** 10.3390/molecules20035050

**Published:** 2015-03-19

**Authors:** Jialong Sun, Yuanming Zhou

**Affiliations:** College of Chemistry and Pharmaceutical Sciences, Qingdao Agricultural University, Qingdao 266109, China; E-Mail: sunjialong6289@163.com

**Keywords:** phenylurea, diacylhydrazine, metaflumizone, insecticidal activity, synthesis

## Abstract

A series of novel phenylurea derivatives were designed and synthesized according to the method of active groups linkage and the principle of aromatic groups bioisosterism in this study. The structures of the novel phenylurea derivatives were confirmed based on ESI-MS, IR and ^1^H-NMR spectral data. All of the compounds were evaluated for the insecticidal activity against the third instars larvae of *Spodoptera exigua* Hiibner, *Plutella xyllostella* Linnaeus, *Helicoverpa armigera* Hubner and *Pieris rapae* Linne respectively, at the concentration of 10 mg/L. The results showed that all of the derivatives displayed strong insecticidal activity. Most of the compounds presented higher insecticidal activity against *S. exigua* than the reference compounds tebufenozide, chlorbenzuron and metaflumizone. Among the synthesized compounds, **3b**, **3d**, **3f**, **4b** and **4g** displayed broad spectrum insecticidal activity.

## 1. Introduction

Benzoylphenyl ureas (BPUs), acting as insect growth regulators (IGRs) and affecting the larval stages of most insects by blocking or inhibiting the synthesis of chitin, have been rapidly developed since the first benzoylphenylurea, diflubenzuron, was commercialized in 1972 [1,2]. BPUs have attracted considerable attention for decades for their unique mode of action coupled with a high degree of activity on target pests and low toxicity to non-target organisms [3,4]. More than 20 BPUs have been developed as IGRs, such as chlorfluazuron, flufenoxuron, triflumuron and chlorbenzuron [5].

Diacylhydrazines have been another of the most important classes of insect growth regulators since the discovery of the *N-tert*-butyl-*N*,*N*'-diacylhydrazines in the mid-1980s by Rohm and Haas Co. [6,7]. They affect the ecdysone receptor and lead to lethal premature molting. For their unique mode of action with high insecticidal selectivity, lower toxicity to vertebrates and simple structure, diacylhydrazines have attracted considerable attention [8,9]. Among nonsteroidal ecdysone agonists, *N*-*tert*-butyl-*N*'-(4-ethylbenzoyl)-3,5-dimethylbenzoylhydrazine (tebufenozide) was the first introduced to market [10,11]. At present, a series of diacylhydrazines, such as methoxyfenozide, halofenozide and chromafenozide have already been widely applied as pesticides in the agrochemical field.

Metaflumizone, which was discovered by Nihon Nohyaku in the early 1990s, is a novel sodium channel blocker insecticide. It belongs to the new class of semicarbazone insecticides and its structure contains an acylhydrazone moiety. It provides good control of most of the economically important lepidopterous pests and other orders pests including Siphonaptera, Diptera, Hymenoptera and Coleoptera. Meanwhile, it presents low risk to non-target organisms as well as humans and the environment. Moreover, insect strains which were resistant to the organophosphates, imidacloprid and carbamates have not showed cross-resistance to metaflumizone. Therefore, metaflumizone has a great potential in Integrated Pest Management(IPM) strategy and resistance management [12,13,14].

**Figure 1:**
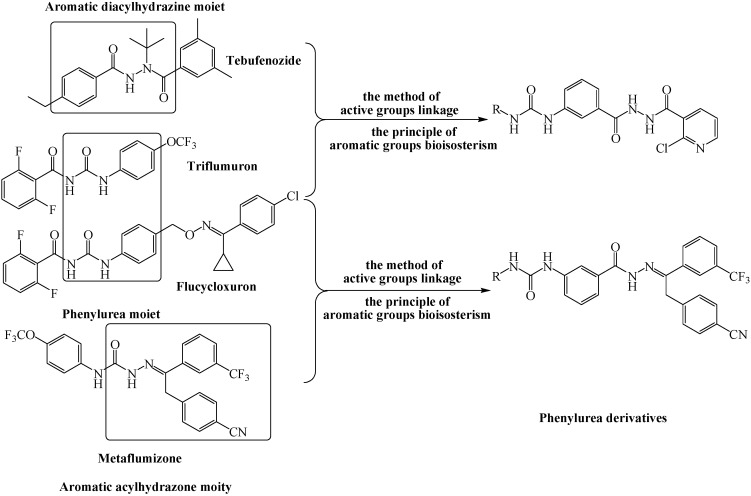
Design of the skeleton of phenylurea derivatives.

An abundance of insects belonging to the order Lepidoptera, such as the diamondback moth *(Helicoverpa armigera* Hubner), beet armyworm (*Spodoptera exigua* Hiibner), cabbage caterpillar (*Pieris rapae* Linne) and cotton bollworm (*Plutella xyllostella* Linnaeus) *etc.*, are among the most damaging pests for crops all over the world. Because they cause enormous agricultural production losses, various insecticides have been used to control Lepidoptera pests. These insecticides brought great benefits, but they have caused negative effects such as toxicity to non-target organisms, including mammals, and environmental pollution. Moreover insecticide resistance has strengthened yearly [15]. Therefore, researchers have to develop novel, low toxicity, highly efficient, friendly environmental insecticides.

**Scheme 1:**
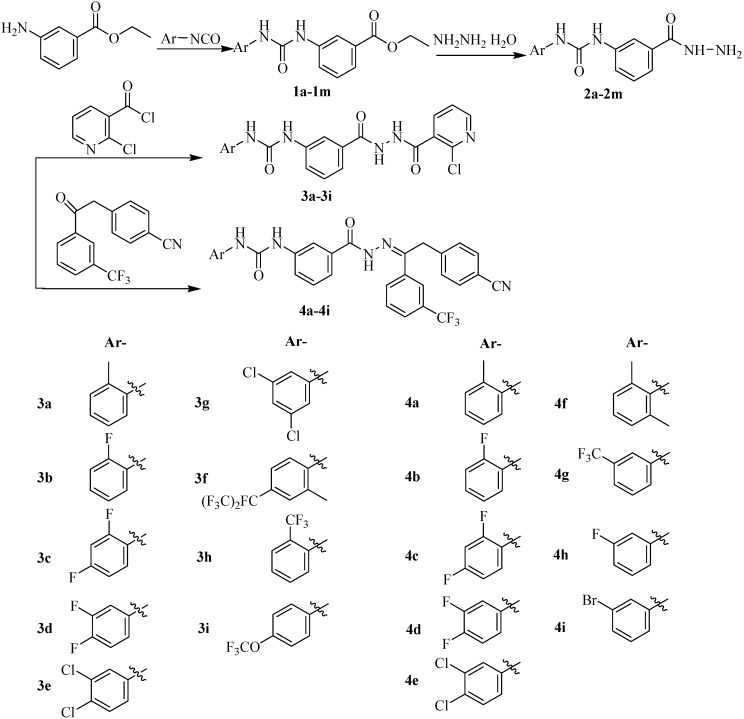
Synthesis of phenylurea derivatives.

In view on the facts above, we sought to incorporate a phenylurea unit with an aromatic diacylhydrazine and acylhydrazone moiety, respectively, according to the method of active groups linkage and the principle of aromatic groups bioisosterism (Figure 1), and eighteen such phenylurea derivatives were then designed and synthesized (Scheme 1). Moreover the new compounds’ insecticidal activities against the third instars larvae of diamondback moth, beet armyworm, cabbage caterpillar and cotton bollworm, were evaluated. We expected that by this approach the combination of the critical components could further strengthen the biological activity of phenylurea derivatives.

## 2. Results and Discussion

### 2.1. Chemistry

As indicated in Scheme 1, eighteen phenylurea derivatives **3a**–**3i**, **4a**–**4i** were successfully synthesized. The raw materials 3-aminobenzoic acid ethyl ester and a substituted phenyl isocyanate (Ar-NCO) were dissolved in toluene and refluxed to give ethyl 3-(3-substituted phenylureido) benzoates **1a**–**1m**. Then with alcohol as solvent, 3-(3-substituted phenylureido)benzohydrazides **2a**–**2m** were produced by the reaction of compounds **1a**–**1m** with hydrazine hydrate. Subsequently, compounds **2a**–**2m** reacted with 2-chloronictinoyl chloride in tetrahydrofuran at room temperature to provide phenylurea derivatives, *N*'-[3-(3-substituted phenylureido)benzoyl]-*N*-(2-chloro)nicotino-hydrazides **3a**–**3i**. Meanwhile, compounds **2a**–**2m** also reacted with 2-(4-nitrilo)benzyl-1-(3-trifluoromethyl)phenyl ketones in tetrahydrofuran at room temperature to give others phenylurea derivatives, N-substituted phenylureido benzoyl-1-(3-trifluoromethyl)phenyl-2-(4-cyano) phenyl ethanone hydrazones **4a**–**4i**. The structures of all the phenylurea derivatives were confirmed by ^1^H-NMR, IR, ESI-MS and MP (Scheme 1).

### 2.2. Insecticidal Activities

As displayed in Table 1, all of the phenylurea derivatives **3a**–**3i**, **4a**–**4i** indicated strong insecticidal activities against the third instars larval of beet armyworm at the concentration of 10 mg/L. Most of the synthesized compounds showed higher insecticidal activity than the references chlorbenzuron, tebufenozide and metaflumizone at the 72 h time point. Among the synthesized compounds, the mortality of the compounds **3g**, **3i**, **4d** and **4e** exceeded 90% at 72 h. Moreover the mortality of compound **4g** reached 100%. Meanwhile the data of Table 1 shows that the mortality of the compounds indicated a positive correlation with administration time.

**Table 1:** Insecticidal activities of the phenylurea derivatives against the third instars larval of beet armyworm (Concentration of 10 mg/L).

Compound	Mortality (%)			Compound	Mortality (%)		
	24 h	48 h	72 h		24 h	48 h	72 h
**3a**	12.50	45.83	83.33	**4a**	25.00	41.67	87.50
**3b**	8.33	37.50	87.50	**4b**	12.50	37.50	75.00
**3c**	4.17	37.50	83.33	**4c**	20.83	45.83	79.17
**3d**	12.50	45.83	87.50	**4d**	50.00	70.83	91.67
**3e**	16.67	50.00	87.50	**4e**	41.67	75.00	95.83
**3f**	8.33	41.67	87.50	**4f**	41.67	66.67	87.50
**3g**	20.83	66.67	95.83	**4g**	45.83	75.00	100.0
**3h**	8.33	41.67	83.33	**4h**	16.67	37.50	79.17
**3i**	16.67	62.50	91.67	**4i**	20.83	45.83	83.33
Tebufenozide	4.17	20.83	41.67	Chlorbenzuron	12.50	16.67	45.83
Metaflumizone	37.50	66.67	75.00				

The data in Table 2 showed the mortality of the phenylurea derivatives **3a**–**3i**, **4a**–**4i** against the third instars larval of diamondback moth (*P. xyllostella*), cotton bollworm (*H. armigera*) and cabbage worm (*P. rapae*) at the concentration of 10 mg/L after 72 h. The data presented that all the phenylurea derivatives showed strong insecticidal activity against diamondback moth, cotton bollworm and cabbage worm. Among the synthesized compounds, **3b**, **3d**, **3f**, **4b** and **4g** displayed broad spectrum insecticidal activity.

**Table 2:** Insecticidal activities of phenylurea derivatives (Concentration of 10 mg/L, 72 h).

Compound	Mortality(%)		
	*P. xyllostella*	*H. armigera*	*P. rapae*
**3a**	87.50	91.67	91.67
**3b**	91.67	95.83	95.83
**3c**	87.50	91.67	91.67
**3d**	91.67	95.83	95.83
**3e**	91.67	87.50	95.83
**3f**	95.83	91.67	95.83
**3g**	83.33	83.33	100.0
**3h**	83.33	87.50	91.67
**3i**	95.83	83.33	100.0
**4a**	79.17	87.50	91.67
**4b**	91.67	95.83	95.83
**4c**	83.33	79.17	87.50
**4d**	79.17	87.50	100.0
**4e**	75.00	87.50	100.0
**4f**	87.50	91.67	87.50
**4g**	91.67	95.83	100.0
**4h**	91.67	87.50	87.50
**4i**	83.33	79.17	87.50
Tebufenozide	83.33	83.33	87.50
Chlorbenzuron	87.50	79.17	83.33
Metaflumizone	66.67	37.50	75.00

## 3. Experimental Section

### 3.1. General Procedures

^1^H-NMR spectra were obtained at 500 MHz using an AM-500 spectrometer in DMSO-*d_6_* solution (Bruker, Karlsruhe, Germany). The melting points were determined on WRS-1A type melting point apparatus (Shanghai, China) and uncorrected. IR spectra were measured on a Nicolet IR-200 spectrophotometer (Thermo Electron, Madison, WI, USA) using KBr disks. A Micromass Q-TOF spectrometer (Waters Crop., Manchester, UK) was used to record HR-ESI-MS data. Pre-coated silica gel plates were used for analytical thin layer chromatography (TLC) and spots were visualized with UV (254 nm).Yields were not optimized.

### 3.2. General Procedure for the Preparation of **1a**–**1m**

3-Aminobenzoic acid ethyl ester (50 mmol) was dissolved in toluene (30 mL).With ice-water bath cooling, a substituted phenyl isocyanate (Ar-NCO) (50 mmol) dissolved in toluene (30 mL), was added dropwise. With the water bath, the mixture was reacted at a temperature of 25 °C, for 5 h, and then heated to reflux for 2 h. After cooling to room temperature, the reaction mixture was filtered under vacuum and dried to produce ethyl 3-(3-substituted phenylureido)benzoates **1a**–**1m**, in yields ranging from 85% to 95%.

### 3.3. General Procedure for the Preparation of **2a**–**2m**

Ethyl 3-(3-substituted phenylureido)benzoates **1a**–**1m** (20 mmol) and 80% hydrazine hydrate (100 mmol) were dissolved in ethanol (100 mL). The mixture was stirred and heated under reflux for 4 h. After cooling to room temperature, the reaction mixture was filtered under vacuum. The solid residue was washed with ice-water (50 mL), to give 3-(3-substituted phenylureido)benzohydrazides **2a**–**2m** in yields ranging from 80% to 95%.

### 3.4. General Procedure for the Preparation of **3a**–**3i**

3-(3-Substituted phenylureido)benzohydrazides **2a**–**2m** (4.42 mmol) was dissolved in dried tetrahydrofuran (50 mL) in a 250 mL round bottom flask. With ice water bath cooling, 2-chloronictinoyl chloride (5.7 mmol) dissolved in dried tetrahydrofuran (50 mL), was added into the flask dropwise. The mixture was reacted at room temperature for 17 h, then it was distilled under reduced pressure to remove the tetrahydrofuran. The solid residue was recrystallized from DMF and water (volume ratio, 1:1) to give the target compounds, *N*'-[3-(3-substituted phenylureido)benzoyl]-*N*-(2-chloro)nicotinohydrazides **3a**–**3i**, with yields ranging from 50% to 70%.

### 3.5. General Procedure for the Preparation of **4a**–**4i**

3-(3-substituted phenylureido)benzohydrazides **2a**–**2m** (2 mmol) and 2-(4-nitrilo)benzyl-1-(3-trifluoromethyl)phenyl ketones (2 mmol) were dissolved in a mixture of methanol (15 mL) and *n*-hexane (2 mL) in a 100 mL round bottom flask and heated to 60 °C with a water bath. After adding three drops of trifluoroacetic acid, the mixture was stirred and reacted for 6 h. The reaction mixture was filtered while hot and the solid obtained was washed with 15 mL methanol, then dried to give the target compounds, *N*-(3-substituted phenylureido)benzoyl-1-(3-trifluoromethyl)phenyl-2-(4-cyano)-phenyl ethanone hydrazones **4a**–**4i**, in yields ranging from 60% to 75%. All eighteen phenylurea derivatives **3a**–**3i**, **4a**–**4i** were novel and the physical and spectral data for these compounds are listed below.

*N'-[3-(3-(2-Methyl)phenyl)ureido]benzoyl-N-(2-chloro)nicotinohydrazide* (**3a**): Yellow solid, yield 63.6%, m.p.203.0~203.9 °C. ^1^H-NMR (DMSO-*d_6_*) δ: 10.68, 10.60 (each s, H, -CONHNHCO-), 9.24 (s, 1H, NH), 8.55 (dd, *J* = 4.8, 1.8 Hz, 1H), 7.96–8.02 (overlap, 4H), 7.84 (d, *J* = 8.0 Hz, 1H), 7.68 (d, *J* = 7.8 Hz, 1H), 7.58 (dd, *J* = 7.4, 4.9 Hz, 1H), 7.52 (d, *J* = 7.7 Hz, 1H), 7.42 (t, *J* = 7.9 Hz, 1H), 7.19 (d, *J* = 7.4 Hz, 1H), 7.15 (t, *J* = 7.6 Hz, 1H), 6.96 (t, *J* = 7.4 Hz, 1H), 2.25 (s, 3H, -CH_3_). IR (KBr): *ν* 3281, 3148, 3027, 1643, 1588, 1561, 1455, 1334, 1295, 1244, 1182, 994, 752, 693 cm^−1^.

*N'-[3-(3-(2-Fluoro)phenyl)ureido]benzoyl-N-(2-chloro)nicotinohydrazide* (**3b**): Gray white solid, yield 60.7%, decomposed before melting. HR-ESI-MS *m/z*: 428.0908 [M+H]^+^ (calcd. for C_20_H_17_ClFN_5_O_3_, 428.0926). ^1^H-NMR (DMSO-*d_6_*) δ: 10.64 (s, 2H, -CONHNHCO-), 9.29(s, 1H, NH), 8.62 (d, *J* = 2.1 Hz, 1H), 8.55 (dd, *J* = 4.8, 1.8 Hz, 1H), 8.16 (dt, *J* = 8.2, 1.3 Hz, 1H), 8.00 (d, *J* = 2.1 Hz, 1H), 7.98 (dd, *J* = 7.6, 1.8 Hz, 1H), 7.67 (dd, *J* = 8.0, 1.8 Hz, 1H), 7.58 (dd, *J* = 7.5, 4.8 Hz, 1H), 7.55 (d, *J* = 7.8 Hz, 1H), 7.44 (t, *J* = 7.9 Hz, 1H), 7.24 (ddd, *J* = 11.5, 8.2, 1.1 Hz, 1H), 7.15 (t, *J* = 7.7 Hz, 1H), 7.03 (ddt, *J* = 7.3, 5.2, 1.5 Hz, 1H). IR (KBr): *ν* 3566, 3394, 3297, 3211, 3078, 3011, 1647, 1588, 1561, 1491, 1451, 1405, 1299, 1252, 1193, 806, 752, 646 cm^−1^.

*N'-[3-(3-(2,4-Difluoro)phenyl)ureido]benzoyl-N-(2-chloro)nicotinohydrazide* (**3c**): Gray white solid, yield 58.3%, m.p. 184.2~185.9 °C. HR-ESI-MS *m/z*: 446.0808 [M+H]^+^ (calcd. for C_20_H_15_ClF_2_N_5_O_3_, 446.0826). ^1^H-NMR (DMSO-*d_6_*) δ: 10.68, 10.60 (each s, 1H, -CONHNHCO-), 9.21 (s, 1H, NH), 8.56 (d, *J* = 2.1 Hz, 1H), 8.55 (dd, *J* = 4.8, 1.9 Hz, 1H), 8.09 (dt, *J* = 9.2, 6.1 Hz, 1H), 7.98 (s, 1H), 7.95 (s, 1H), 7.66 (dd, *J* = 8.0, 1.3 Hz, 1H), 7.57 (dd, *J* = 7.5, 4.8 Hz, 1H), 7.55 (d, *J* = 7.8 Hz, 1H), 7.43 (t, *J* = 7.9 Hz, 1H), 7.33 (ddd, *J* = 11.6, 8.9, 2.8 Hz, 1H), 7.06 (t, *J* = 7.8 Hz, 1H). IR (KBr): *ν* 3539, 3304, 3172, 3035, 1721, 1663, 1600, 1553, 1432, 1401, 1190, 1139, 1096, 845, 802, 740 cm^−1^.

*N'-[3-(3-(3,4-Difluoro)phenyl)ureido]benzoyl-N-(2-chloro)nicotinohydrazide* (**3d**): Yellow solid, yield 52.5%, m.p. 214.9~215.2 °C. HR-ESI-MS *m/z*: 446.0829 [M+H]^+^ (calcd. for C_20_H_15_ClF_2_N_5_O_3_, 446.0826). ^1^H-NMR (DMSO-*d_6_*) δ: 10.67, 10.59 (each s, 1H, -CONHNHCO-), 8.97 (s, 2H, -NHCONH-), 8.55 (dd, *J* = 4.8, 1.8 Hz, 1H), 7.96–8.02 (overlap, 2H), 7.64–7.70 (overlap, 2H), 7.58 (dd, *J* = 7.5, 4.8 Hz, 1H), 7.55 (d, *J* = 7.8 Hz, 1H), 7.43 (t, *J* = 7.9 Hz, 1H), 7.36 (d, *J* = 10.4 Hz, 1H), 7.15 (m, 1H). IR (KBr): *ν* 3344, 3249, 3091, 1685, 1648, 1561, 1515, 1490, 1436, 1403, 1208, 905, 860, 748, 673 cm^−1^.

*N'-[3-(3-(3,4-Dichloro)phenyl)ureido]benzoyl-N-(2-chloro)nicotinohydrazide* (**3e**): Gray white solid, yield 54.9%, m.p. 194.2~194.7 °C. HR-ESI-MS *m/z*: 478.0235 [M+H]^+^ (calcd. for C_20_H_15_Cl_3_N_5_O_3_, 478.0235). ^1^H-NMR (DMSO-*d_6_*) δ: 10.68, 10.60 (each s, 1H, -CONHNHCO-), 9.06, 9.02 (each s, 1H, -NHCONH-), 8.55 (dd, *J* = 4.8, 1.9 Hz, 1H), 8.01 (s, 1H), 7.99 (dd, *J* = 7.5, 1.9 Hz, 1H), 7.91 (d, *J* = 2.4 Hz, 1H), 7.66 (d, *J* = 8.0 Hz, 1H), 7.58 (dd, *J* = 7.6, 4.8 Hz, 1H), 7.56 (d, *J* = 7.8 Hz, 1H) 7.53 (d, *J* = 8.8 Hz, 1H), 7.43 (t, *J* = 7.9 Hz, 1H), 7.35 (dd, *J* = 8.8, 2.4 Hz, 1H). IR (KBr): *ν* 3277, 3236, 3093, 3010, 1653, 1581, 1536, 1473, 1401, 1378, 1292, 1223, 1132, 906, 808, 748 cm^−1^.

*N'-[3-(3-(3,5-Dichloro)phenyl)ureido]benzoyl-N-(2-chloro)nicotinohydrazide* (**3f**): Gray white solid, yield 65.6%, m.p. 223.6~224.5 °C. HR-ESI-MS *m/z*: 478.0235 [M+H]^+^ (calcd. for C_20_H_15_Cl_3_N_5_O_3_, 478.0235). ^1^H-NMR (DMSO-*d_6_*) δ: 10.68, 10.60 (each s, 1H, -CONHNHCO-), 9.12, 9.11 (each s, 1H, -NHCONH-), 8.56 (dd, *J* = 4.8, 1.9 Hz, 1H), 8.01 (s, 1H), 7.99 (dd, *J* = 7.5, 1.8 Hz, 1H), 7.95 (s, 1H), 7.66 (d, *J* = 7.9 Hz, 1H), 7.54–7.60 (overlap, 3H), 7.45 (t, *J* = 7.9 Hz, 1H), 7.17 (t, *J* = 1.8 Hz, 1H). IR (KBr): *ν* 3362, 3308, 3168, 1725, 1659, 1604, 1584,1538, 1484, 1392, 1304, 1281, 1246, 1195, 1100, 1063, 820, 739, 665 cm^−1^.

*N'-[3-(3-(4-Ditrifluoromethylmethyl)phenyl)ureido]benzoyl-N-(2-chloro)nicotinohydrazide* (**3g**): white powder, yield 62.4%, m.p. 196.76~198.1 °C. ^1^H-NMR (DMSO-*d_6_*) δ: 10.64 (s, 2H, -CONHNHCO-), 9.45 (s, 1H, NH), 8.55 (dd, *J* = 4.8, 1.8 Hz, 1H), 8.24 (d, *J* = 8.8 Hz, 1H), 8.22 (s, 1H), 8.02 (s, 1H), 7.99 (dd, *J* = 7.5, 1.8 Hz, 1H), 7.69 (dd, *J* = 7.9, 1.2 Hz, 1H), 7.58 (dd, *J* = 7.5, 4.8 Hz, 1H), 7.55 (d, *J* = 7.8 Hz, 1H), 7.43–7.49 (overlap, 3H), 2.35 (s, 3H, -PhCH_3_). IR (KBr): *ν* 3254, 3005, 1662, 1613, 1584, 1533, 1401, 1306, 1272, 1218, 1178, 1135, 1106, 977, 888, 811, 754 cm^−1^.

*N'-[3-(3-(2-Trifluoromethyl)phenyl)ureido]benzoyl-N-(2-chloro)nicotinohydrazide* (**3h**): Yellow solid, yield 68.7%, m.p. 199.3~199.6 °C. HR-ESI-MS *m/z*: 478.0889 [M+H]^+^ (calcd. for C_21_H_16_ClF_3_N_5_O_3_, 478.0888). ^1^H-NMR (DMSO-*d_6_*) δ: 10.66, 10.62 (each s, 1H, -CONHNHCO-), 9.58 (s, 1H, NH), 8.55 (dd, *J* = 4.8, 1.2 Hz, 1H), 8.13 (s, 1H), 7.94–8.02 (m, overlap, 2H), 7.62–7.72 (m, overlap, 3H), 7.53–7.60 (m, overlap, 2H), 7.434 (t, *J* = 7.9 Hz, 1H), 7.30 (t, *J* = 7.5 Hz, 2H). IR (KBr): *ν* 3572, 3435, 3303, 3216, 3083, 3012, 1652, 1590, 1544, 1507, 1453, 1407, 1316, 1279, 1179, 1109, 1059, 1038, 905, 806, 764, 690, 648 cm^−1^.

*N'-[3-(3-(4-Trifluoromethoxyl)phenyl)ureido]benzoyl-N-(2-chloro)nicotinohydrazide* (**3i**): white powder, yield 59.1%, m.p. 233.6~234.4 °C. HR-ESI-MS *m/z*: 494.0839 [M+H]^+^ (calcd. for C_21_H_16_ClF_3_N_5_O_4_, 494.0837). ^1^H-NMR (DMSO-*d_6_*) δ: 10.67, 10.60 (s, 1H, -CONHNHCO-), 8.99, 8.97 (each s, 1H, -NHCONH-), 8.55 (dd, *J* = 4.8, 1.8 Hz, 1H), 7.97–8.02 (m, overlap, 2H), 7.94 (s, 1H), 7.67 (d, *J* = 7.7 Hz, 1H), 7.58 (d, *J* = 8.6 Hz, 1H), 7.54 (d, *J* = 7.3 Hz, 1H), 7.43 (t, *J* = 7.9 Hz, 1H), 7.30 (d, *J* = 8.7 Hz, 2H). IR (KBr): *ν* 3373, 3299, 3182, 3033, 2929, 2855, 1723, 1660, 1611, 1590, 1507, 1478, 1399, 1279, 1154, 1096, 827, 744, 682, 636 cm^−1^.

*N-[3-(3-(2-Methyl)phenyl)ureido]benzoyl-1-(3-trifluoromethyl)phenyl-2-(4-cyano)phenylethanone hydrazone* (**4a**): White powder, yield 70.0%, m.p. 208.3~208.7 °C. HR-ESI-MS *m/z*: 556.195 [M+H]^+^ (calcd for C_31_H_25_F_3_N_5_O_2_, 556.1955). ^1^H-NMR (DMSO-*d_6_*) δ: 11.34 (s, 1H, PhCONH-), 9.24 (s, 1H, ‑NHCONH-), 8.17 (s, 1H), 8.07 (s, 1H), 7.96 (s, 1H), 7.91 (s, 1H), 7.83 (d, *J* = 7.7 Hz, 1H), 7.77 (d, *J* = 8.0 Hz, 2H), 7.73 (s, 1H), 7.55–7.70 (overlap, 2H), 7.38–7.45 (overlap, 3H), 7.34 (d, *J* = 7.7 Hz, 1H), 7.12–7.22 (overlap, 2H), 6.96 (t, *J* = 7.2 Hz, 1H), 4.60 (s, 2H, -CH_2_-), 2.24 (s, 3H, -CH_3_). IR (KBr):*ν* 3336, 3307, 3216, 3050, 2958, 2229, 1652, 1590, 1548, 1486, 1432, 1349, 1258, 1171, 1117, 1080, 893, 764, 702, 644, 545 cm^−1^.

*N-[3-(3-(2-Fluoro)phenyl)ureido]benzoyl-1-(3-trifluoromethyl)phenyl-2-(4-cyano)phenylethanone hydrazone* (**4b**): White powder, yield 63.4%, m.p. 239.0~239.6 °C. HR-ESI-MS *m/z*: 560.1706 [M+H]^+^ (calcd for C_30_H_22_F_3_N_5_O_2_, 560.1704). ^1^H-NMR (DMSO-*d_6_*) δ: 11.34 (s, 1H, PhCONH-), 9.27, 8.59 (each s, 1H, -NHCONH-), 8.15 (t, *J* = 8.0 Hz, 2H), 8.07 (s, 1H), 7.92 (s, 1H), 7.77 (d, *J* = 8.2 Hz, 2H), 7.72 (s, 1H), 7.63 (s, 2H), 7.35–7.45 (m, overlap, 4H), 7.25 (dd, *J* = 10.7, 8.4 Hz, 1H), 7.15 (t, *J* = 7.4 Hz, 1H), 7.01 (dd, *J* = 12.6, 6.2 Hz, 1H), 4.60 (s, 2H, -CH_2_-). IR (KBr): *ν* 3328, 3257, 3104, 2925, 2224, 1723, 1669, 1590, 1544, 1519, 1436, 1295, 1129, 1071, 752, 694, 553 cm^−1^.

*N-[3-(3-(2,4-Difluoro)phenyl)ureido]benzoyl-1-(3-trifluoromethyl)phenyl-2-(4-cyano)phenylethanone hydrazone* (**4c**): White powder, yield 67.5%, m.p. 243.6~243.7 °C. HR-ESI-MS *m/z*: 578.1612 [M+H]^+^ (calcd for C_30_H_21_F_5_N_5_O_2_, 578.1610).^1^H-NMR (DMSO-*d_6_*) δ: 11.33 (s, 1H, PhCONH-), 9.21, 8.54 (each s, 1H, -NHCONH-), 8.17 (s, 1H), 8.02–8.12 (overlap, 2H), 7.91 (s, 1H), 7.77 (d, *J* = 8.2 Hz, 2H), 7.73 (s, 1H), 7.63 (s, 2H), 7.35–7.45 (overlap, 4H), 7.30 (m, 1H), 7.06 (t, *J* = 8.2 Hz, 1H), 4.60 (s, 2H, -CH_2_-). IR (KBr): *ν* 3535, 3308, 3257, 3187,2577, 1678, 1565, 1397, 1186, 1131, 1096, 1064, 842, 744, 611 cm^−1^.

*N-[3-(3-(3,4-Difluoro)phenyl)ureido]benzoyl-1-(3-trifluoromethyl)phenyl-2-(4-cyano)phenylethanone hydrazone* (**4d**): White powder, yield 69.6%, m.p. 243.2~243.5 °C. HR-ESI-MS *m/z*: 578.1606 [M+H]^+^ (calcd for C_30_H_21_F_5_N_5_O_2_, 578.1610). ^1^H-NMR (DMSO-*d_6_*) δ: 11.32 (s, 1H, PhCONH-), 8.96, 8.94 (each s, 1H, -NHCONH-), 8.17 (s, 1H), 8.06 (s, 1H), 7.90 (s, 1H), 7.77 (d, *J* = 8.2 Hz, 2H), 7.73 (s, 1H), 7.59–7.70 (m, overlap, 3H), 7.30–7.45 (m, overlap, 5H), 7.14 (d, *J* = 7.9 Hz, 1H), 4.60 (s, 2H, -CH_2_-). IR (KBr): *ν*3336, 3316, 3258, 3101, 2226, 1718, 1663, 1593, 1550, 1510, 1432, 1209, 1131, 1072, 1022, 869, 689, 552 cm^−1^.

*N-[3-(3-(3,4-Dichloro)phenyl)ureido]benzoyl-1-(3-trifluoromethyl)phenyl-2-(4-cyano)phenylethanone hydrazone* (**4e**): White powder, yield 72.4%, m.p. 245.4~245.8 °C. HR-ESI-MS *m/z*: 610.1021 [M+H]^+^ (calcd. for C_30_H_21_Cl_2_F_3_N_5_O_2_, 610.1019).^1^H-NMR (DMSO-*d_6_*) δ: 11.33 (s, 1H, PhCONH-), 9.07, 9.05 (each s, 1H, -NHCONH-), 8.18 (s, 1H), 8.06 (s, 1H), 7.92 (s, 1H), 7.89 (d, *J* = 2.2 Hz, 1H), 7.77 (d, *J* = 8.2 Hz, 2H), 7.73 (s, 1H), 7.64 (s, 2H), 7.52 (d, *J* = 8.8 Hz, 1H), 7.31–7.45 (m, overlap, 5H), 4.60 (s, 2H, -CH_2_-). IR (KBr): *ν* 3325, 3299, 3096, 3033, 2228, 1716, 1659, 1590, 1518, 1473, 1430, 1378, 1326, 1289, 1192, 1132, 1069, 854, 811, 745, 691, 545 cm^−1^.

*N-[3-(3-(2,6-Dimethyl)phenyl)ureido]benzoyl-1-(3-trifluoromethyl)phenyl-2-(4-cyano)phenyl ethanone hydrazone* (**4f**): White powder, yield 70.4%, m.p. 240.1~240.3 °C. ^1^H-NMR (DMSO-*d_6_*) δ: 11.31 (s, 1H, PhCONH-), 8.96 (s, 1H, -NHCONH-), 8.16 (s, 1H), 8.06 (s, 1H), 7.91 (s, 1H), 7.77 (s, 1H), 7.75 (s, 1H), 7.73 (s, 1H), 7.56–7.69 (overlap, 2H), 7.39 (d, *J* = 8.1 Hz, 2H), 7.36 (d, *J* = 7.9 Hz, 1H), 7.31 (d, *J* = 7.7 Hz, 1H), 7.05–7.11 (overlap, 3H), 4.58 (s, 2H, -CH_2_-), 2.20 (s, 6H, -CH_3_). IR (KBr): *ν* 3295, 3159, 3004, 2227, 1655, 1640, 1607,1595,,1487, 1342, 1307, 1266, 1241, 1176, 800, 770, 745 cm^−1^.

*N-[3-(3-(3-Trifluoromethyl)phenyl)ureido]benzoyl-1-(3-trifluoromethyl)phenyl-2-(4-cyano)phenyl ethanone hydrazone* (**4g**): White powder, yield 68.7%, m.p. 220.0~221.2 °C. HR-ESI-MS *m/z*: 610.1669 [M+H]^+^ (calcd. for C_31_H_22_F_6_N_5_O_2_, 610.1672). ^1^H-NMR (DMSO-*d_6_*) δ: 11.33 (s, 1H, PhCONH-), 9.08, 9.01 (each s, 1H, -NHCONH-), 8.18 (s, 1H), 8.07 (s, 1H), 8.03 (s, 1H), 7.93 (s, 1H), 7.77 (d, *J* = 8.2 Hz, 2H), 7.73 (s, 1H), 7.61–7.70 (overlap, 2H), 7.59 (d, *J* = 8.0 Hz, 1H), 7.52 (t, *J* = 7.9 Hz, 1H), 7.35–7.45 (m, overlap, 4H), 7.32 (d, *J* = 7.6 Hz, 1H), 4.60 (s, 2H, -CH_2_-). IR (KBr): *ν*3336, 3261, 3207, 3093, 2225, 1717, 1663, 1596, 1553, 1514, 1491, 1444, 1330, 1295, 1252, 1197, 1154, 1115, 1068, 869, 795, 752, 693, 670, 552 cm^−1^.

*N-[3-(3-(3-Fluoro)phenyl)ureido]benzoyl-1-(3-trifluoromethyl)phenyl-2-(4-cyano)phenylethanone hydrazone* (**4h**): Yellow powder, yield 62.5%, m.p. 231.7~231.9 °C. HR-ESI-MS *m/z*: 560.1706 [M+H]^+^ (calcd. for C_30_H_22_F_4_N_5_O_2_, 560.1704). ^1^H-NMR (DMSO-*d_6_*) δ: 11.33 (s, 1H, PhCONH-), 8.94 (s, 2H, -NHCONH-), 8.17, 8.07, 7.91 (each s, 1H), 7.77 (d, *J* = 8.2 Hz, 2H), 7.73, 7.65 (each s, 1H), 7.50 (d, *J* = 11.8 Hz, 1H), 7.35–7.45 (m, overlap, 4H), 7.31 (dd, *J* = 15.3, 7.9 Hz, 1H), 7.14 (d, *J* = 7.8 Hz), 6.79 (dt, *J* = 8.4, 1.9 Hz, 1H), 4.60 (s, 2H, -CH_2_-). IR (KBr): *ν*3340, 3311, 3257, 3145, 3100, 2224, 1714, 1664, 1511, 1490, 1436, 1308, 1196, 1071, 1017, 897, 872, 752, 690, 557 cm^−1^.

*N-[3-(3-(3-Bromo)phenyl)ureido]benzoyl-1-(3-trifluoromethyl)phenyl-2-(4-cyano)phenyl ethanone hydrazone* (**4i**): White powder, yield 61.8%, m.p. 232.4~232.8 °C. HR-ESI-MS *m/z*: 620.0906 [M+H]^+^(calcd. for C_30_H_22_BrF_3_N_5_O_2_, 620.0903).^1^H-NMR (DMSO-*d_6_*) δ: 11.33 (s, 1H, PhCONH-), 8.97, 8.93 (each s, 1H, -NHCONH-), 8.18, 8.07, 7.92 (each s, 1H), 7.86 (s, 1H), 7.77 (d, *J* = 8.2 Hz, 2H), 7.74 (s, 1H), 7.64 (s, 2H), 7.39–7.44 (m, overlap, 3H), 7.37 (d, *J* = 7.6 Hz, 1H), 7.33 (d, *J* = 7.4 Hz, 1H), 7.25 (t, *J* = 8.0 Hz, 1H), 7.16 (d, *J* = 7.7 Hz, 1H), 4.60 (s, 2H, -CH_2_-). IR (KBr): *ν*3336, 3303, 3265, 3104, 2224, 1723, 1656, 1590, 1536, 1515, 1478, 1428, 1300, 1196, 1129, 1071, 1017, 993, 901, 856, 752, 694, 553 cm^−1^.

### 3.6. Bioassay for Insecticidal Activities

Wheat leaf discs (0.5 cm × 0.5 cm) were treated with 5 μL solutions made from 5 mg of test sample dissolved in 500 mL of acetone. Tebufenozide, chlorbenzuron and metaflumizone were used as positive controls and acetone was used as negative control. The third instars larvae of beet armyworm were fed with the discs. Cohorts of 24 beet armyworms were treated each time and bioassays were replicated three times. After 24 h, 48 h and 72 h, the numbers of knocked down larvae (symptoms: the larvae were narcotized, the bodies were very soft and immobilized, and response disappeared completely) were recorded respectively [16,17].

Bioassay for insecticidal activities against cotton bollworm, diamondback moth and cabbage worm was performed according to the above method. The third instars larvae of cotton bollworm, diamondback moth and cabbage worm were fed with the discs instead, respectively. The numbers of knocked down larvae after 72 h were recorded. The experiments showed that the mortality of acetone as negative controls was 0%.

## 4. Conclusions

In conclusion, eighteen phenylurea derivatives **3a**–**3i**, **4a**–**4i** have been designed and synthesized according to the method of active groups linkage and the principle of aromatic groups bioisosterism. The insecticidal activities of these novel phenylurea derivatives against the third instars larvae of *S. exigua, H. armigera*, *P. xyllostella* and *P. rapae* were evaluated, at a concentration of 10 mg/L. The bioassay results showed that all of the synthesized compounds displayed high insecticidal activity. Most of them presented higher insecticidal activity against *S. exigua* than the reference compounds tebufenozide, chlorbenzuron and metaflumizone. Among the synthesized compounds, **3b**, **3d**, **3f**, **4b** and **4g** displayed broad spectrum insecticidal activity. The results above have encouraged us to further explore novel phenylurea derivatives as insecticidal agents and this will be reported in future work.

## Supplementary Materials

Supplementary materials can be accessed at: http://www.mdpi.com/1420-3049/20/03/5050/s1.
