# Supplementary material for: Design, Synthesis and Insecticidal Activity of Novel Phenylurea Derivatives
Source: Molecules. 2015 Mar 19;20(3):5050–61. doi: 10.3390/molecules20035050 (PMC6272132; doi:10.3390/molecules20035050)
Supplement: Supplementary file 1 [file molecules-20-05050-s001.pdf]

Supplementary Materials

The ¹H-NMR, MS and IR spectra of phenylurea derivatives (3a–3i, 4a–4i) were listed below:

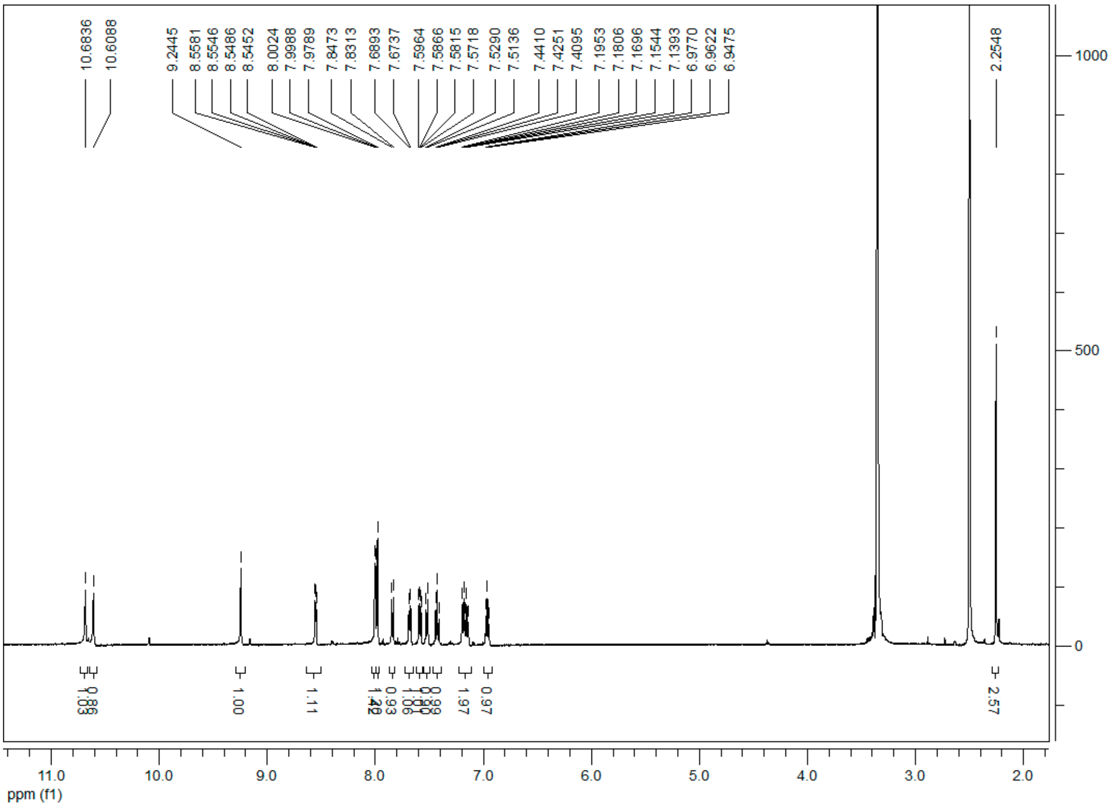

Figure S1. ¹H-NMR of compound **3a** (500 MHz, DMSO-d_6).

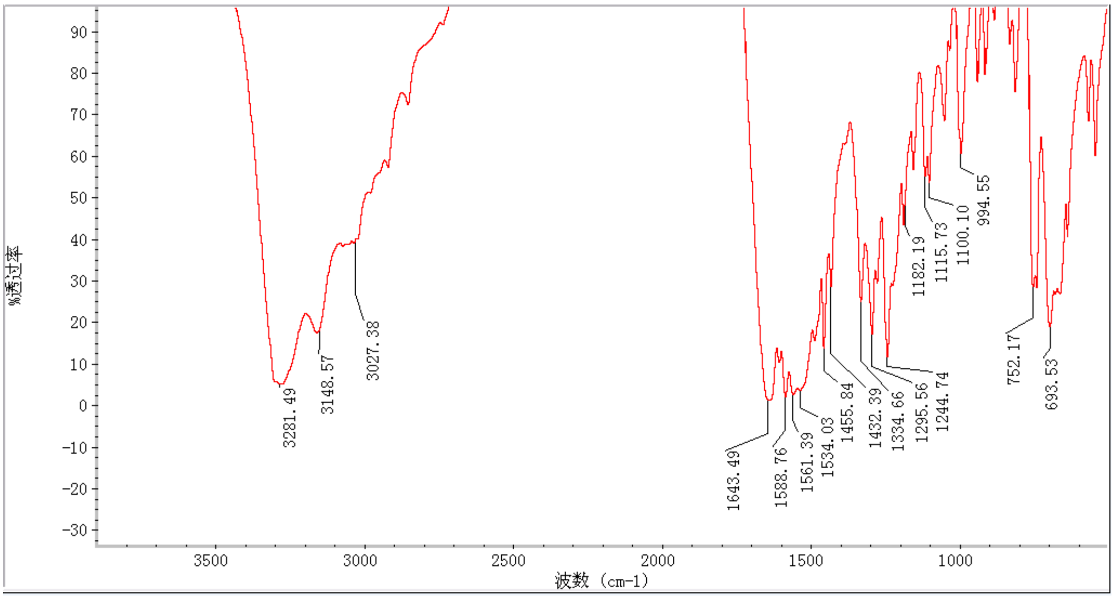

Figure S2. IR of compound **3a**.

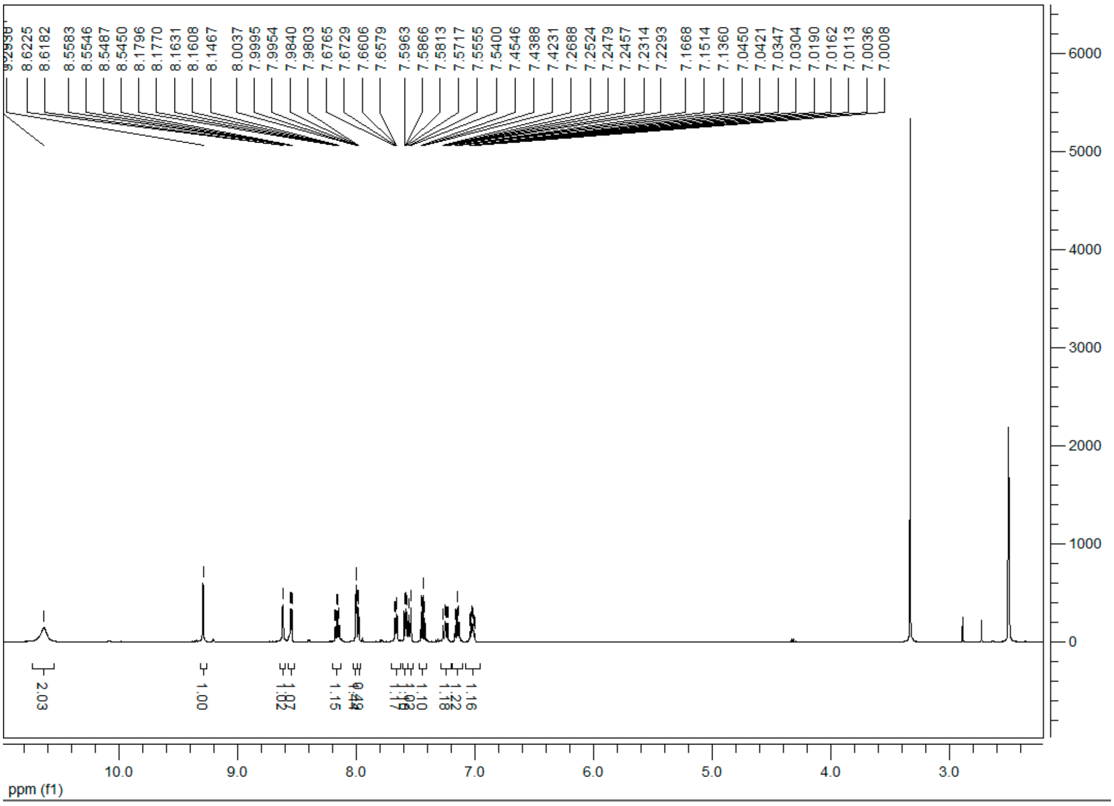

Figure S3. ^{1}H-NMR of compound **3b** (500 MHz, DMSO-*d*$_6$).

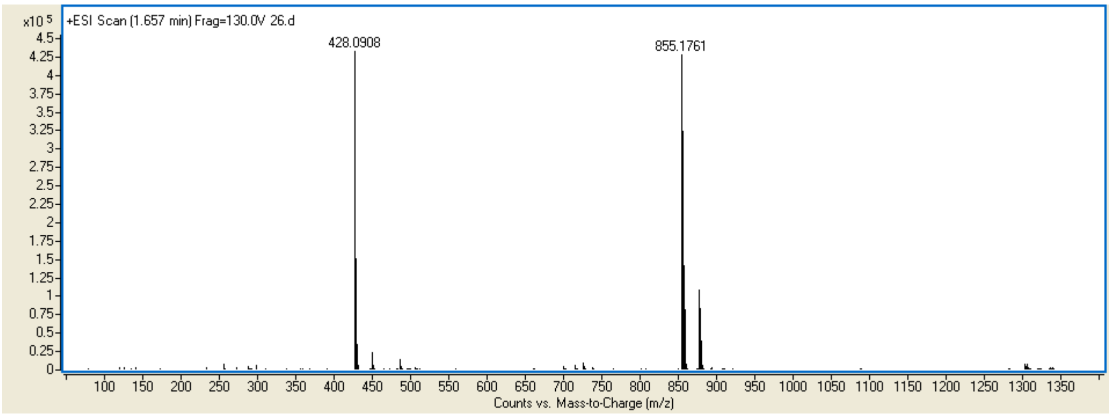

Figure S4. HR-ESI-MS of compound **3b**.

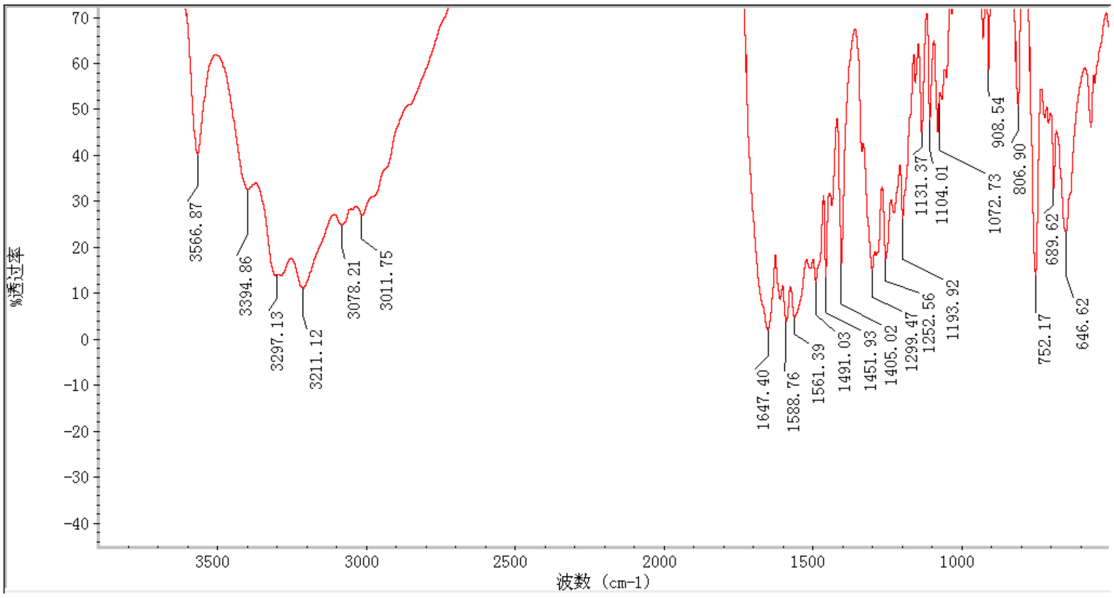

Figure S5. IR of compound **3b**.

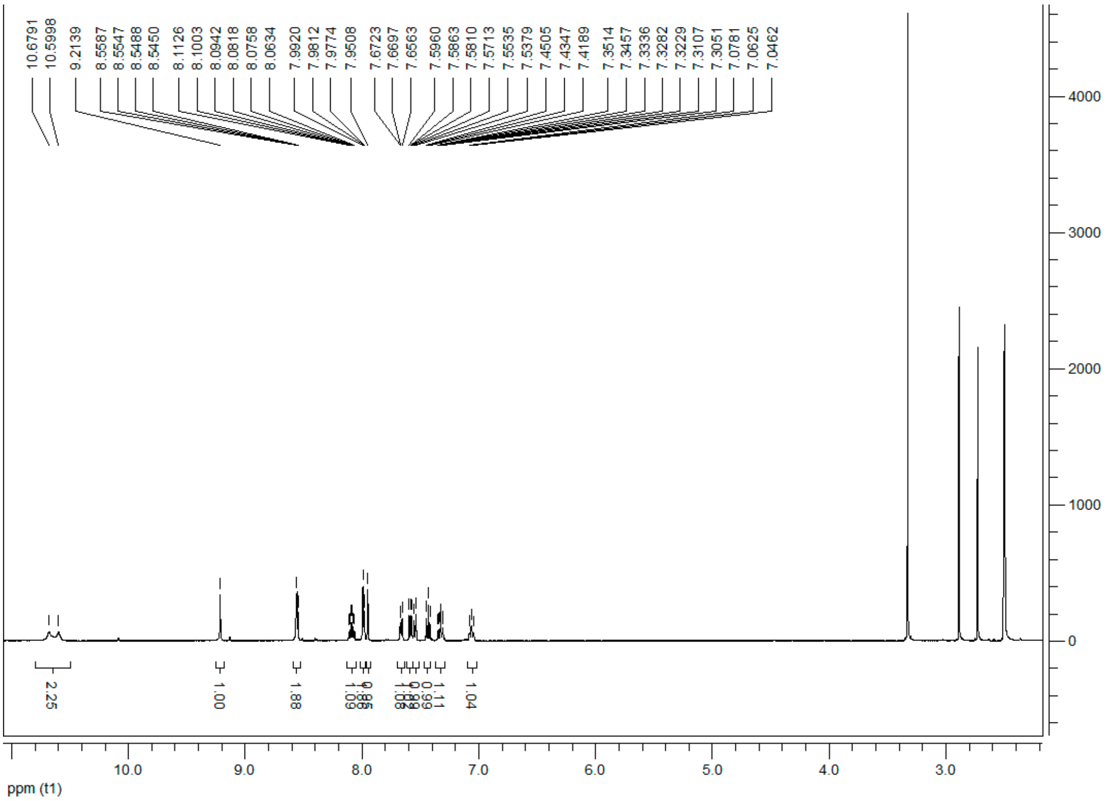

Figure S6. ^{1}H-NMR of compound **3c** (500 MHz, DMSO-d_6).

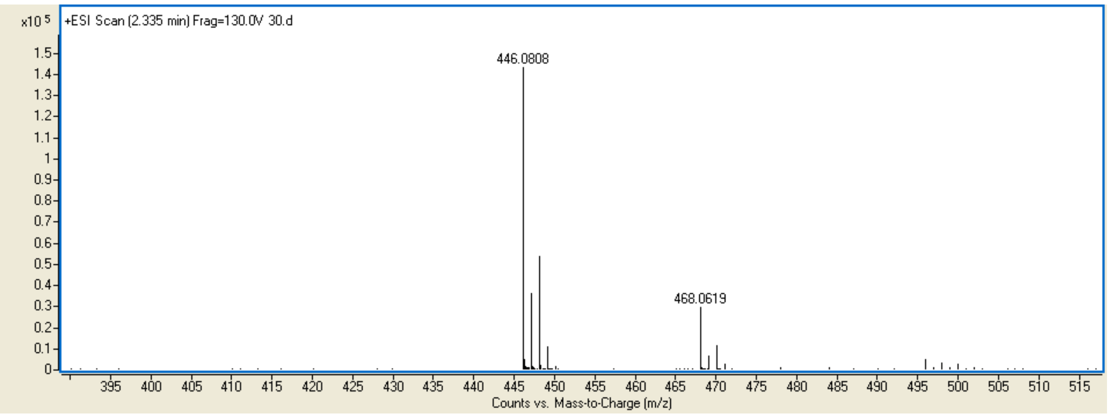

Figure S7. HR-ESI-MS of compound **3c**.

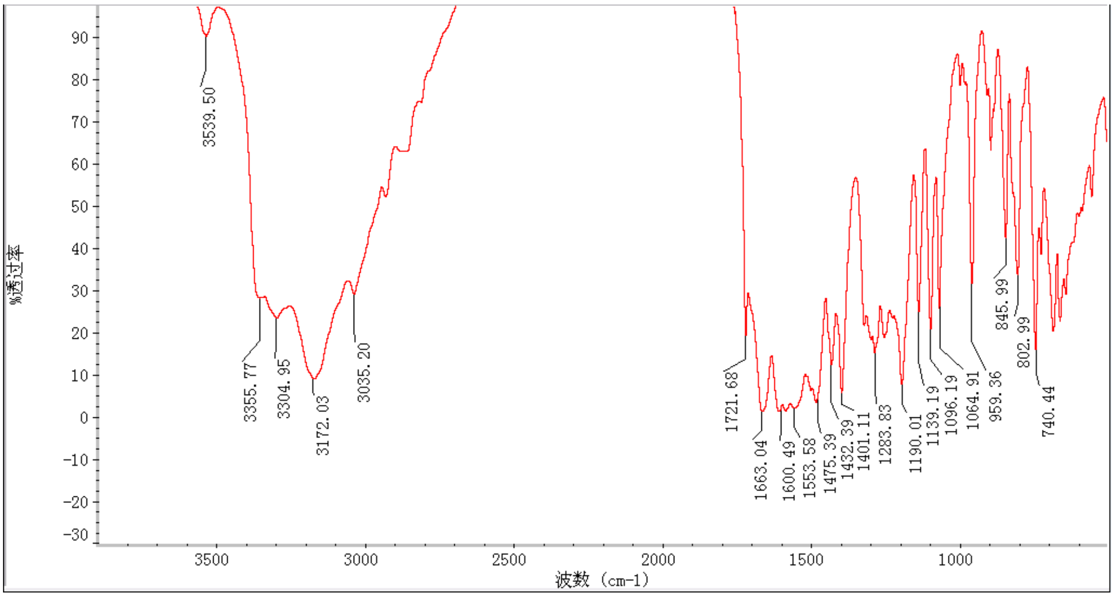

Figure S8. IR of compound **3c**.

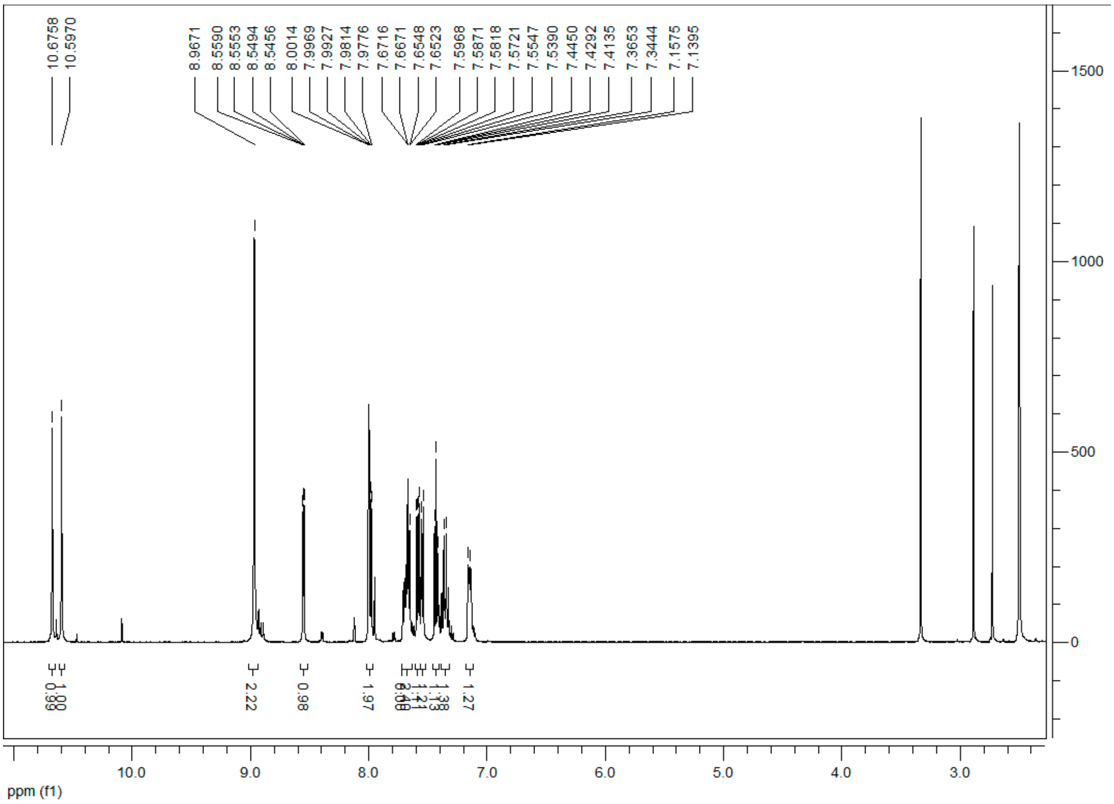

Figure S9. ^{1}H-NMR of compound **3d** (500 MHz, DMSO-d_6).

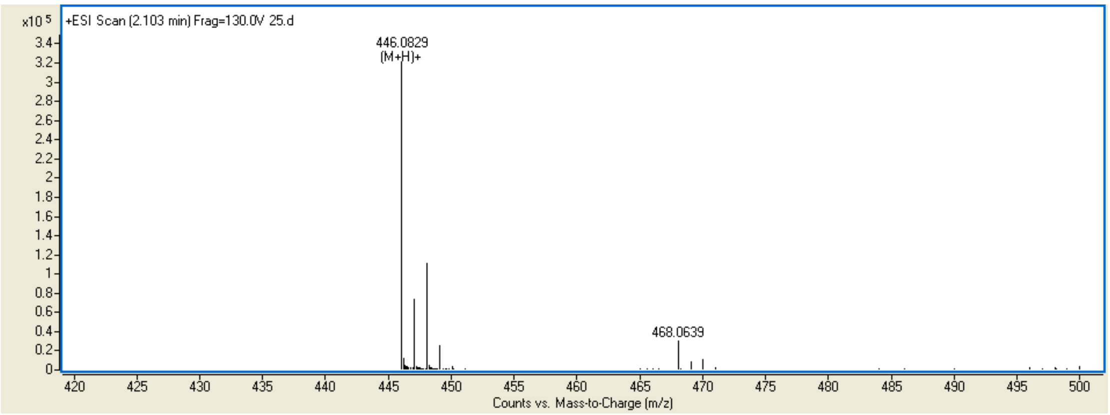

Figure S10. HR-ESI-MS of compound **3d**.

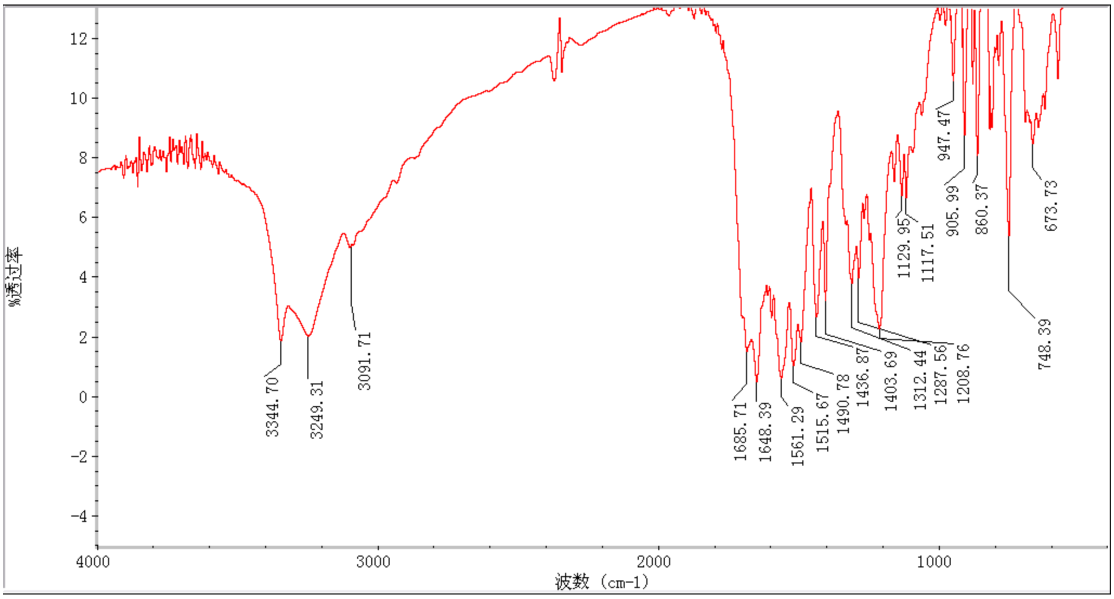

Figure S11. IR of compound **3d**.

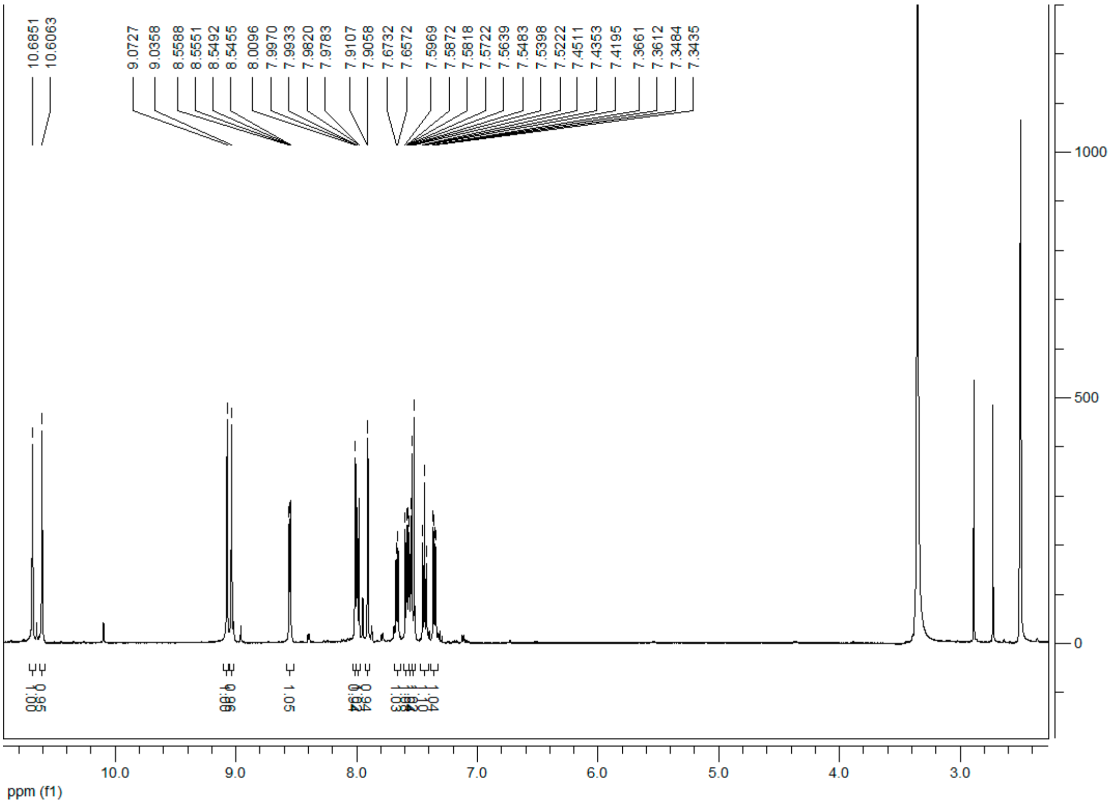

Figure S12. ^{1}H-NMR of compound **3e** (500 MHz, DMSO-d_6).

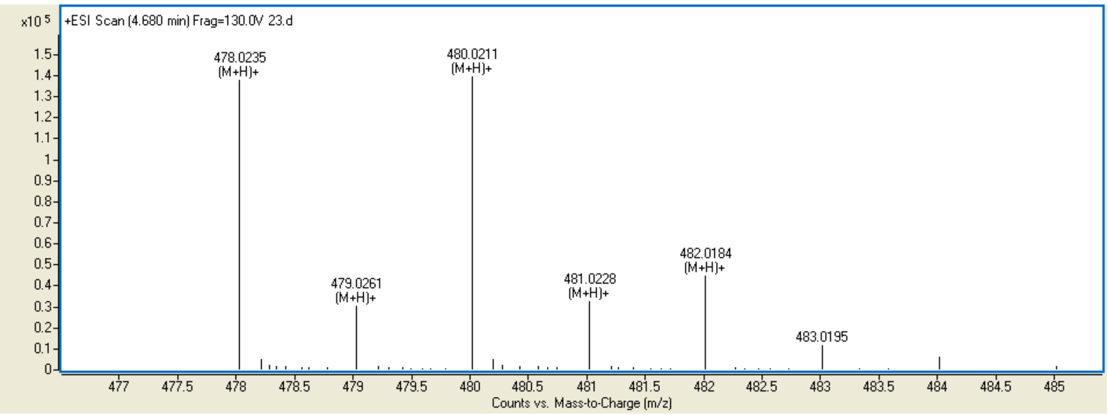

Figure S13. HR-ESI-MS of compound **3e**.

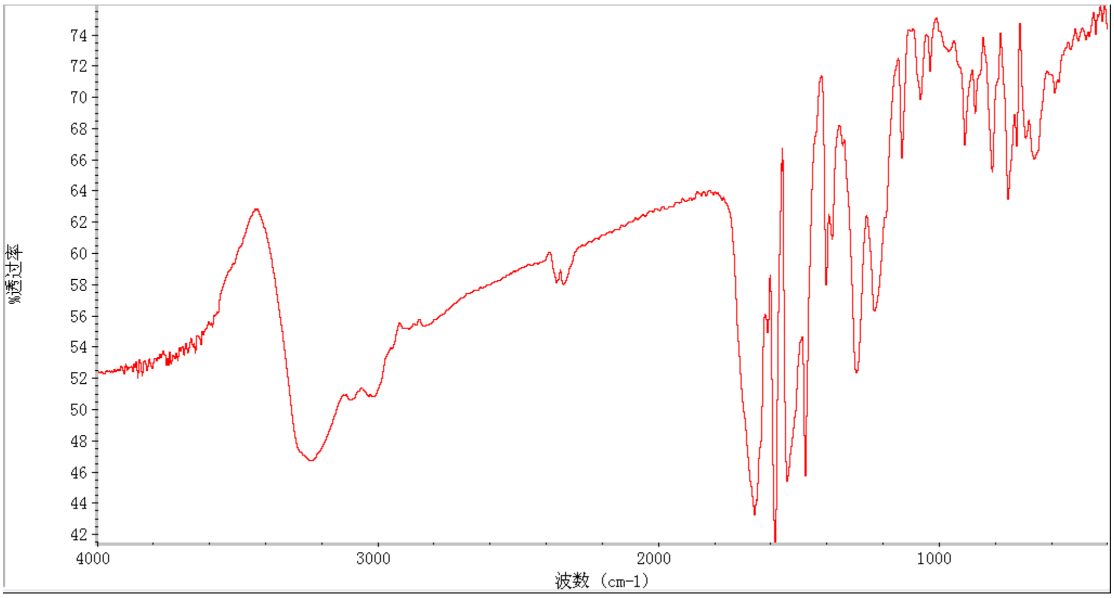

Figure S14. IR of compound **3e**.

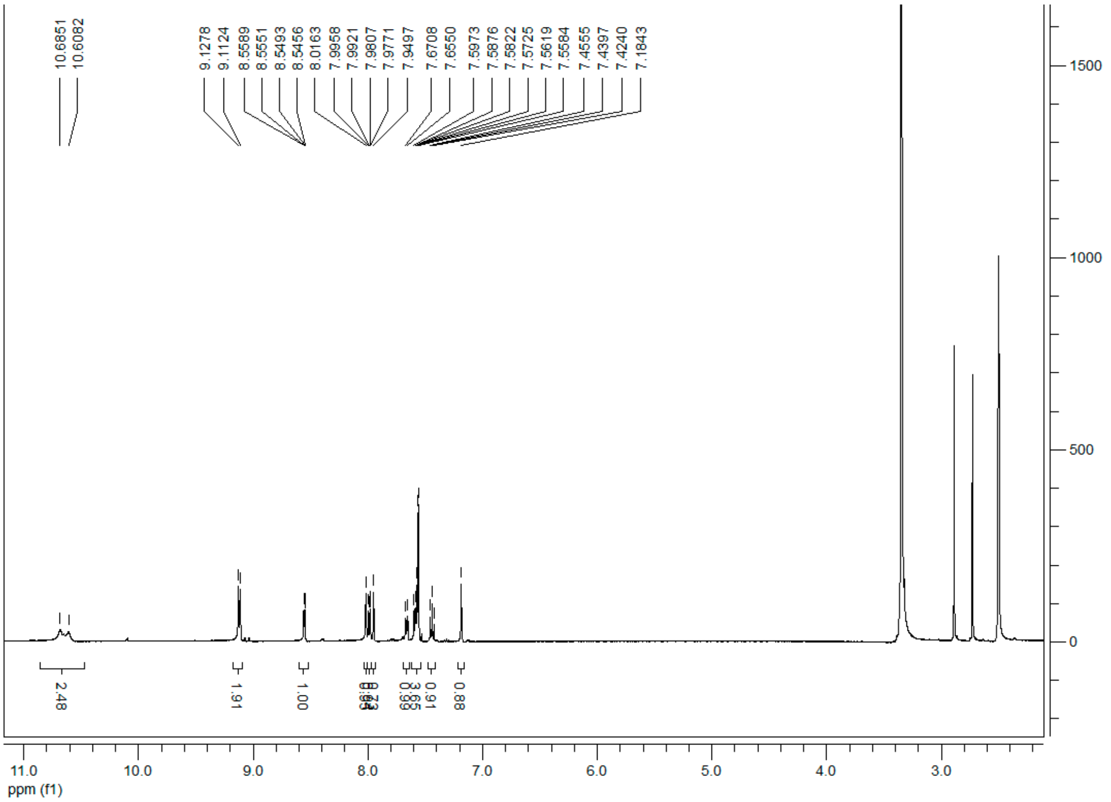

Figure S15. ^{1}H-NMR of compound **3f** (500 MHz, DMSO-*d*$_6$).

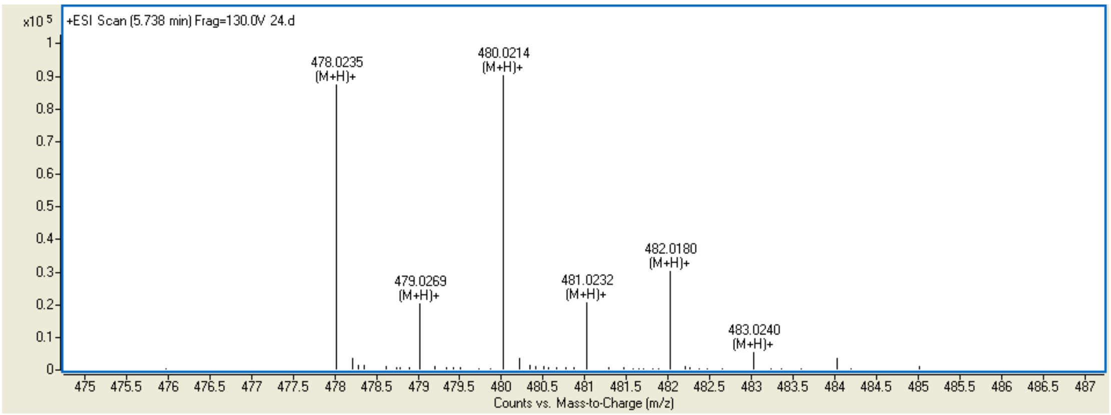

Figure S16. HR-ESI-MS of compound **3f**.

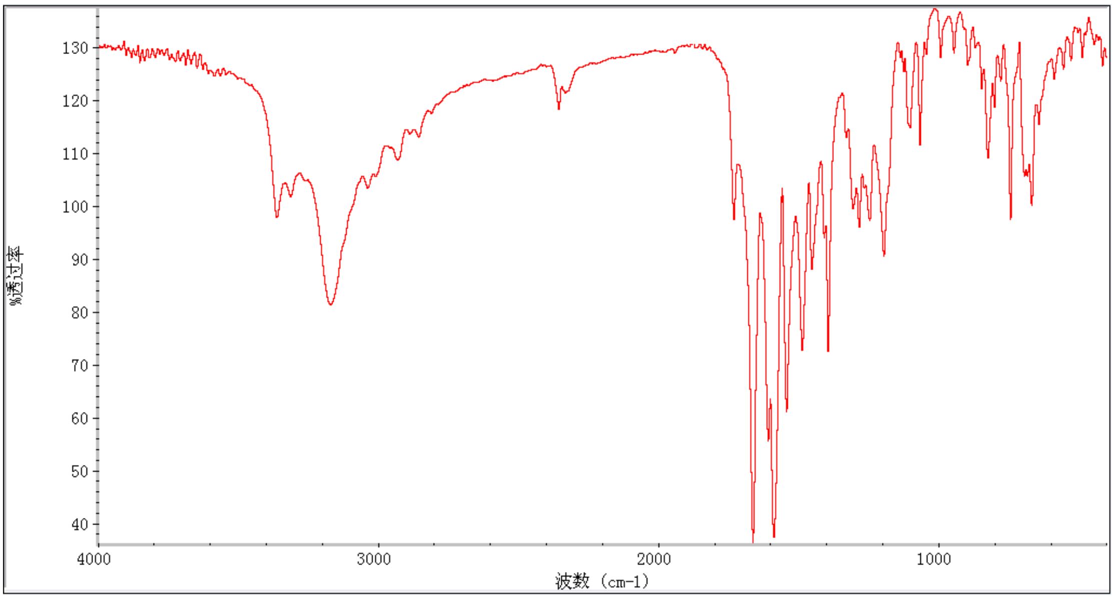

Figure S17. IR of compound **3f**.

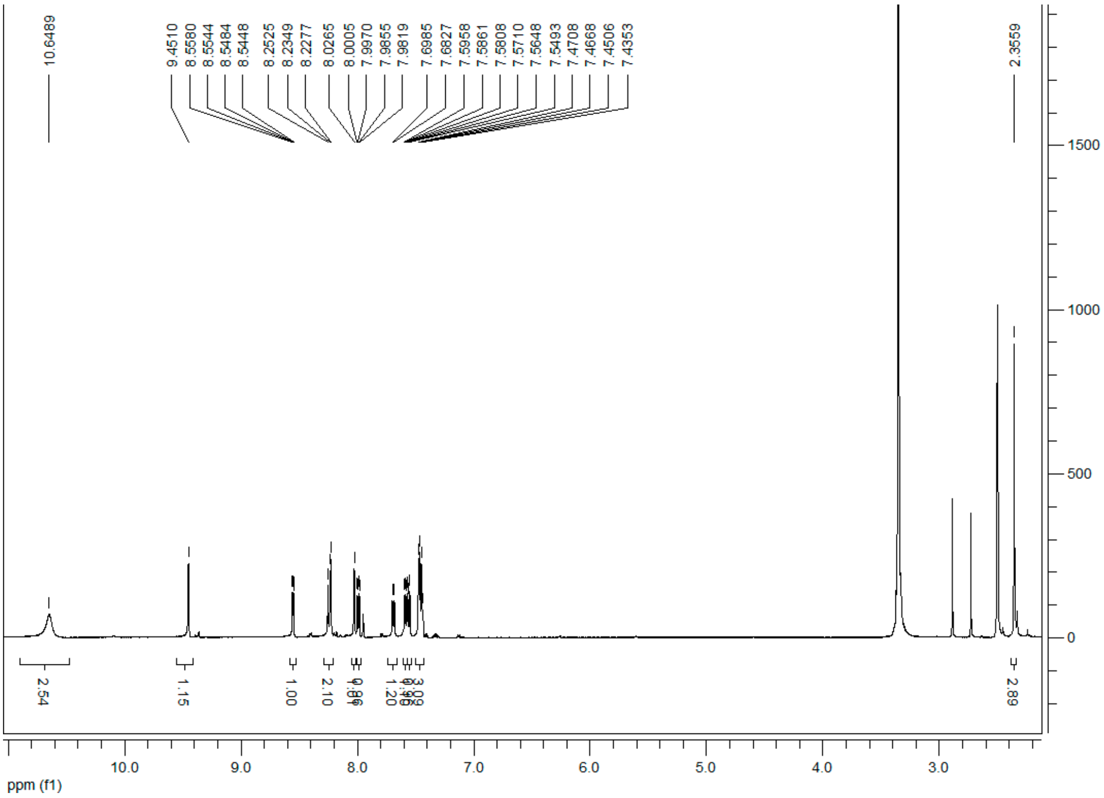

Figure S18. ^{1}H-NMR of compound **3g** (500 MHz, DMSO-*d*6).

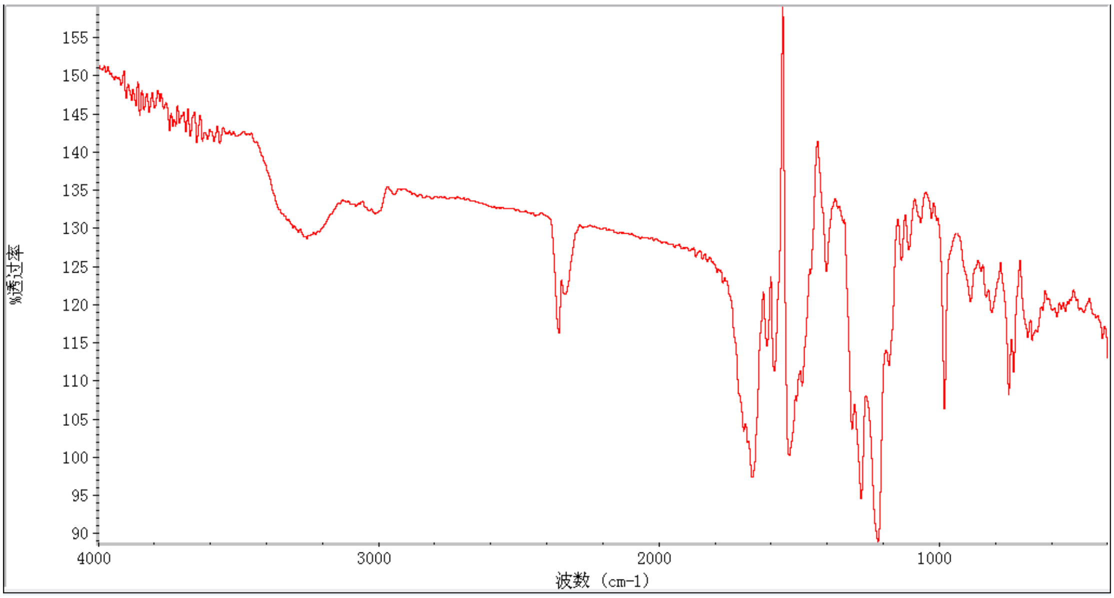

Figure S19. IR of compound **3g**.

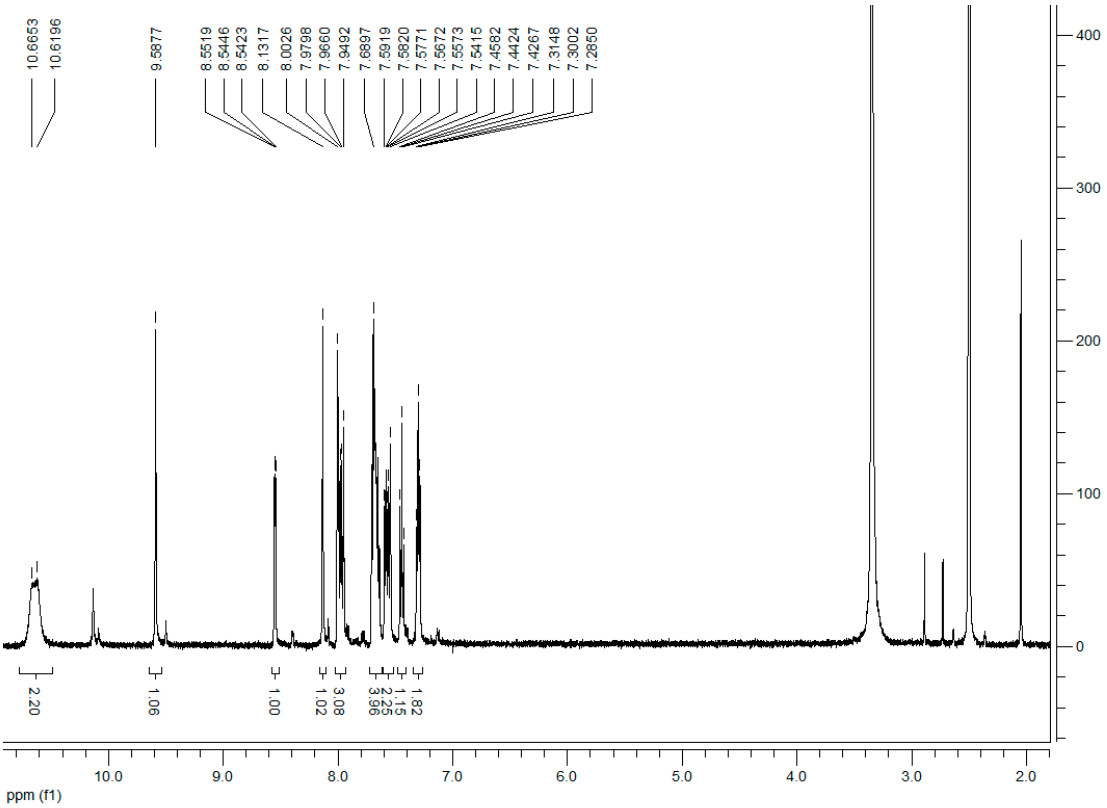

Figure S20. ^{1}H-NMR of compound **3h** (500 MHz, DMSO-d_6).

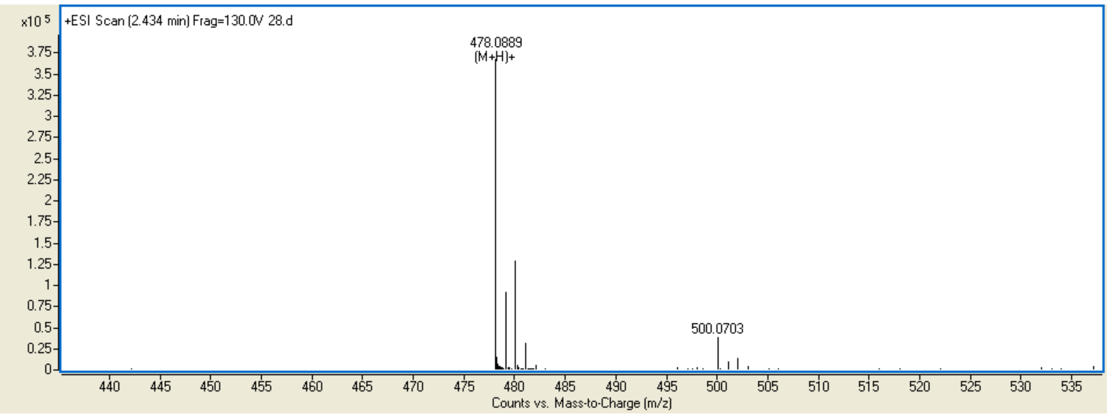

Figure S21. HR-ESI-MS of compound **3h**.

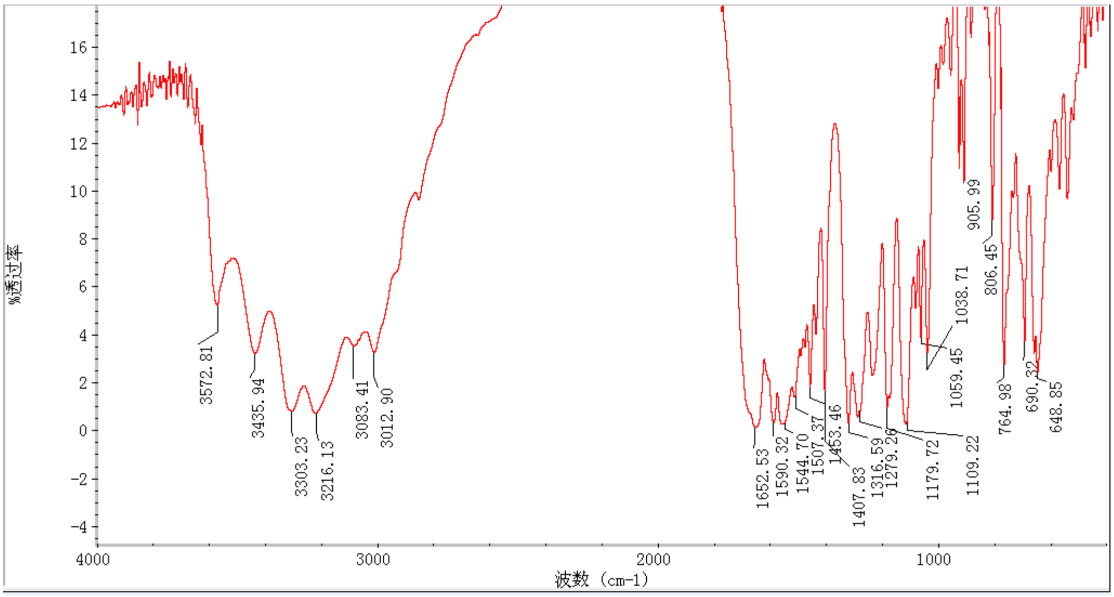

Figure S22. IR of compound **3h**.

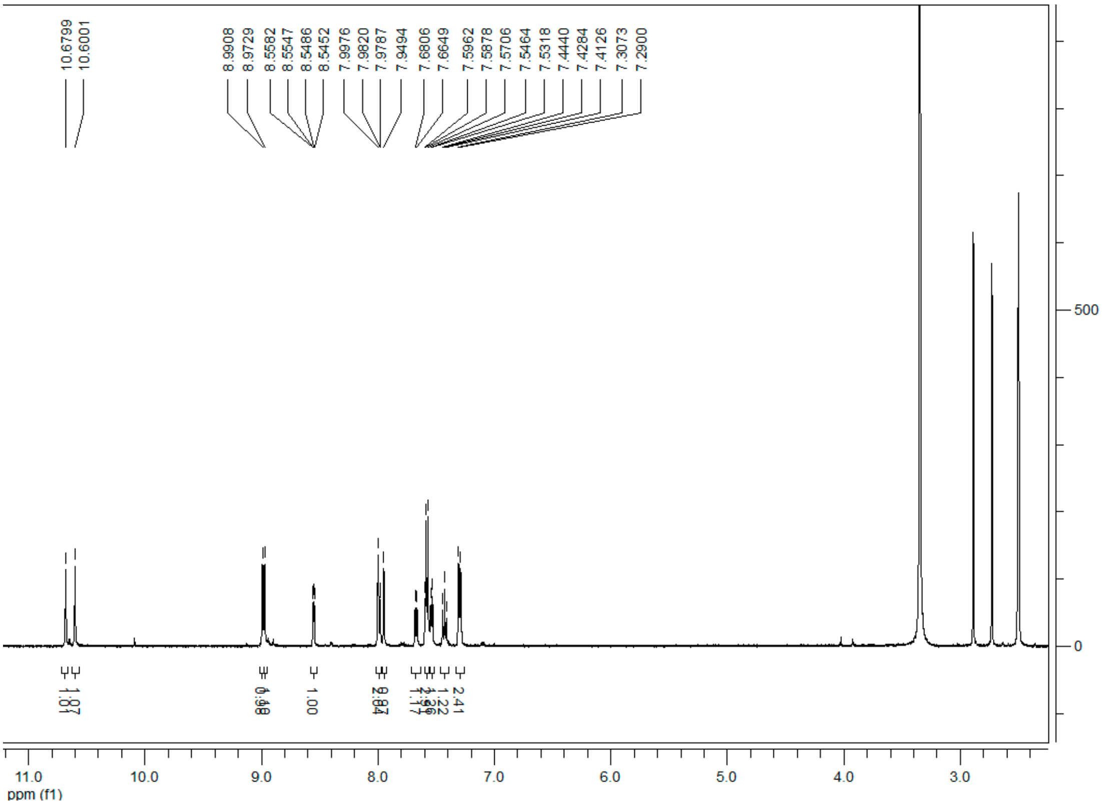

Figure S23. ^{1}H-NMR of compound **3i** (500 MHz, DMSO-*d₆*).

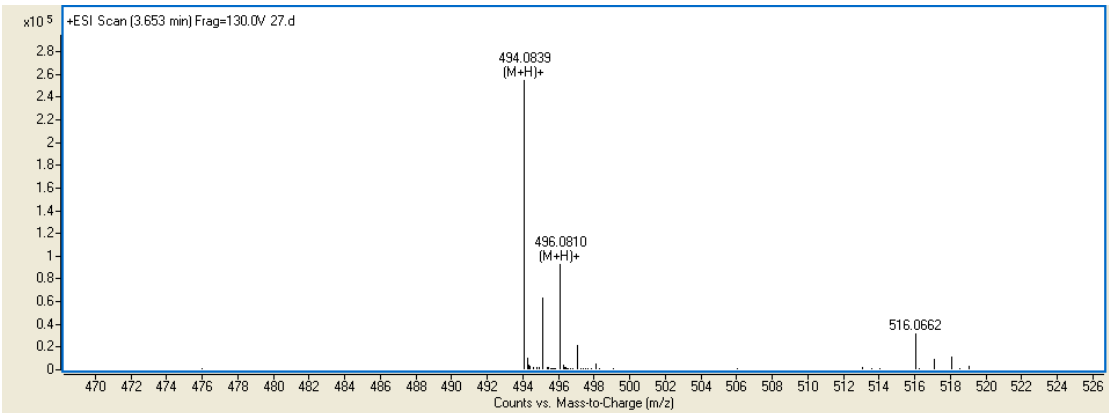

Figure S24. HR-ESI-MS of compound **3i**.

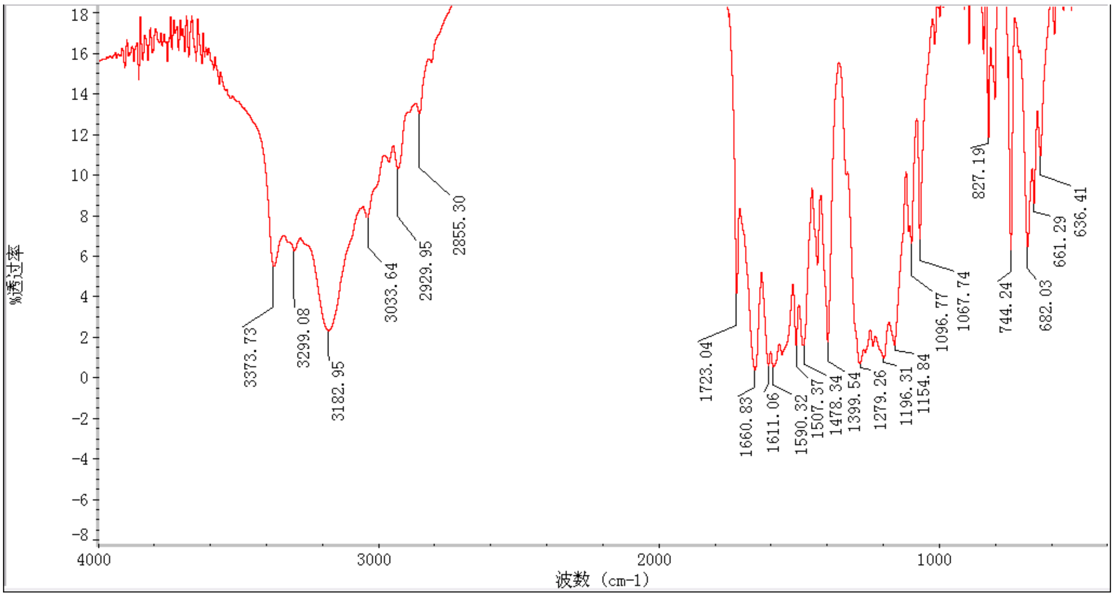

Figure S25. IR of compound **3i**.

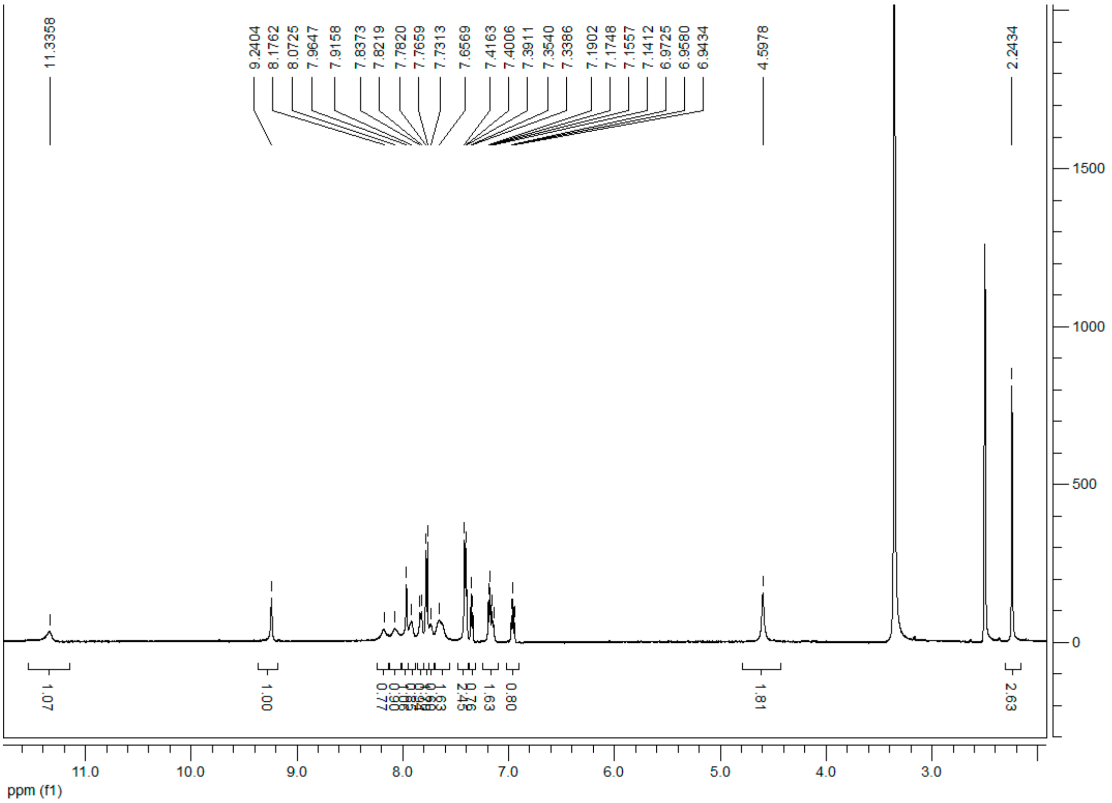

Figure S26. ^{1}H-NMR of compound **4a** (500 MHz, DMSO-d_6).

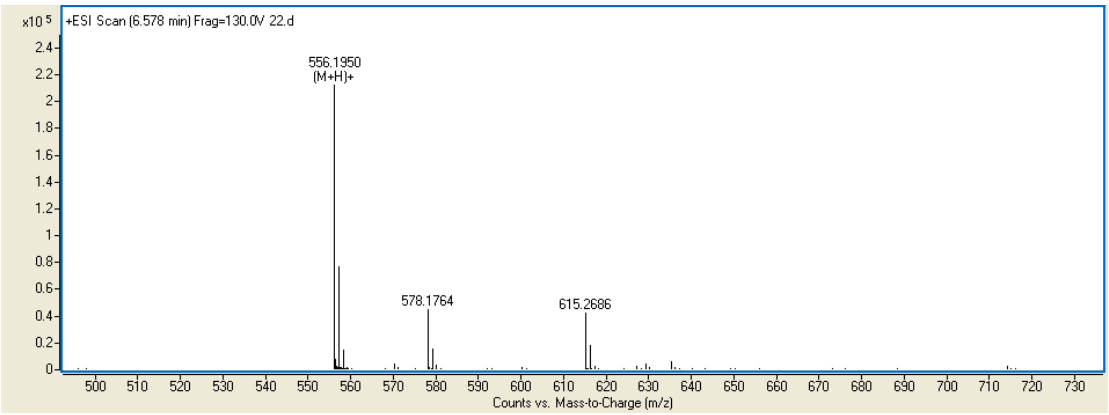

Figure S27. HR-ESI-MS of compound **4a**.

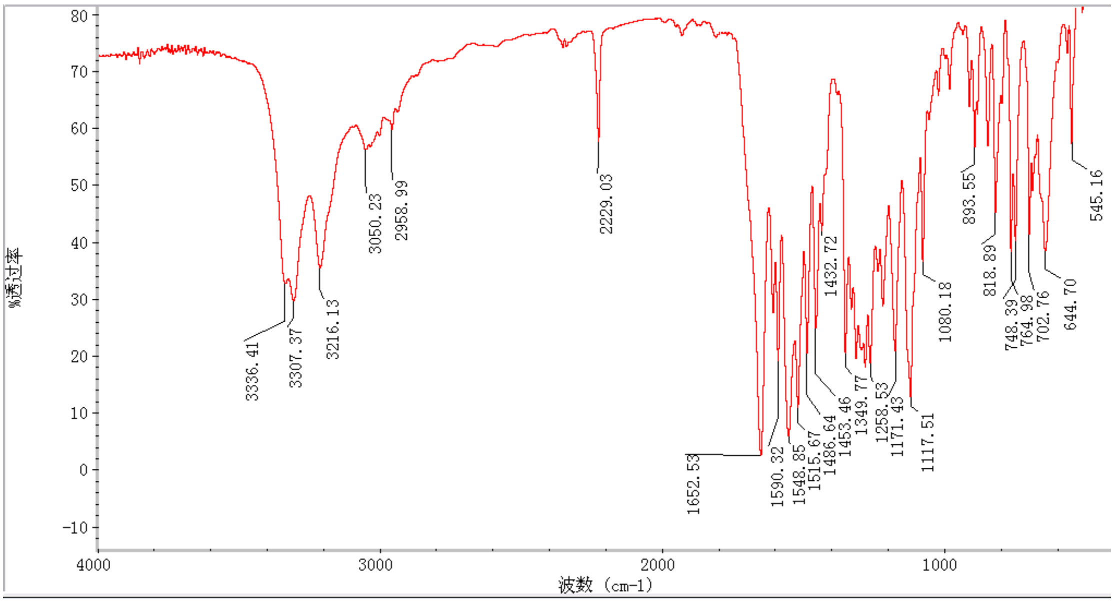

Figure S28. IR of compound **4a**.

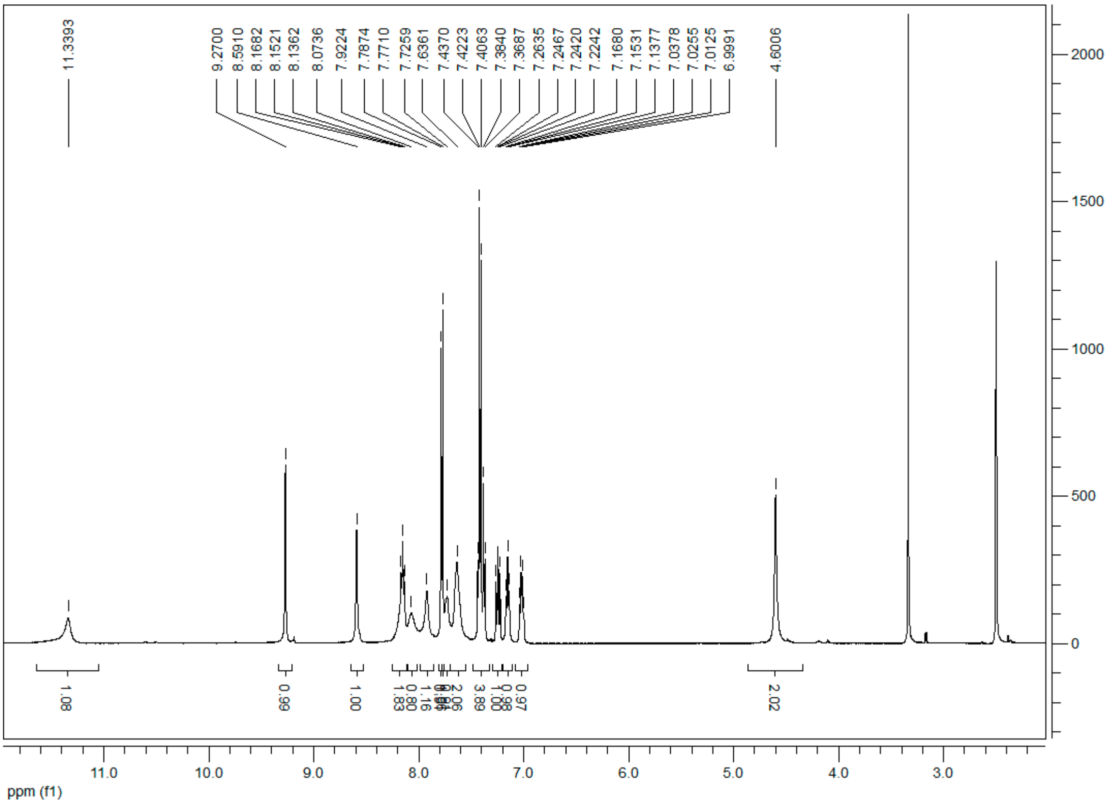

Figure S29. ^{1}H-NMR of compound **4b** (500 MHz, DMSO-d_6).

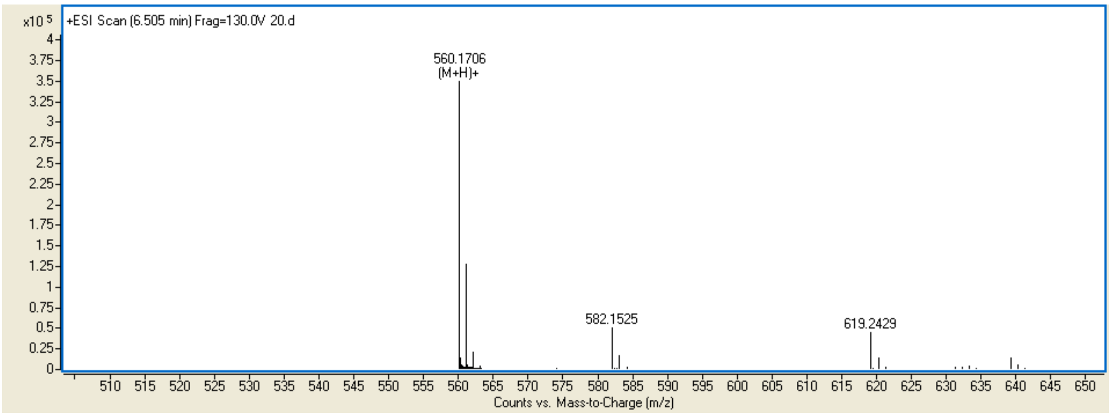

Figure S30. HR-ESI-MS of compound **4b**.

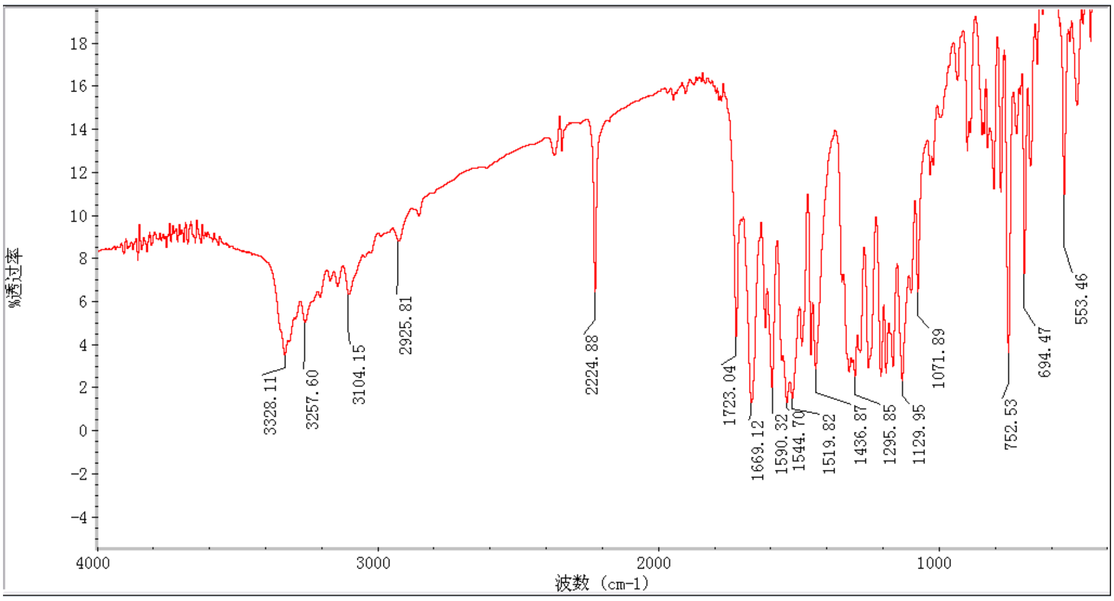

Figure S31. IR of compound **4b**.

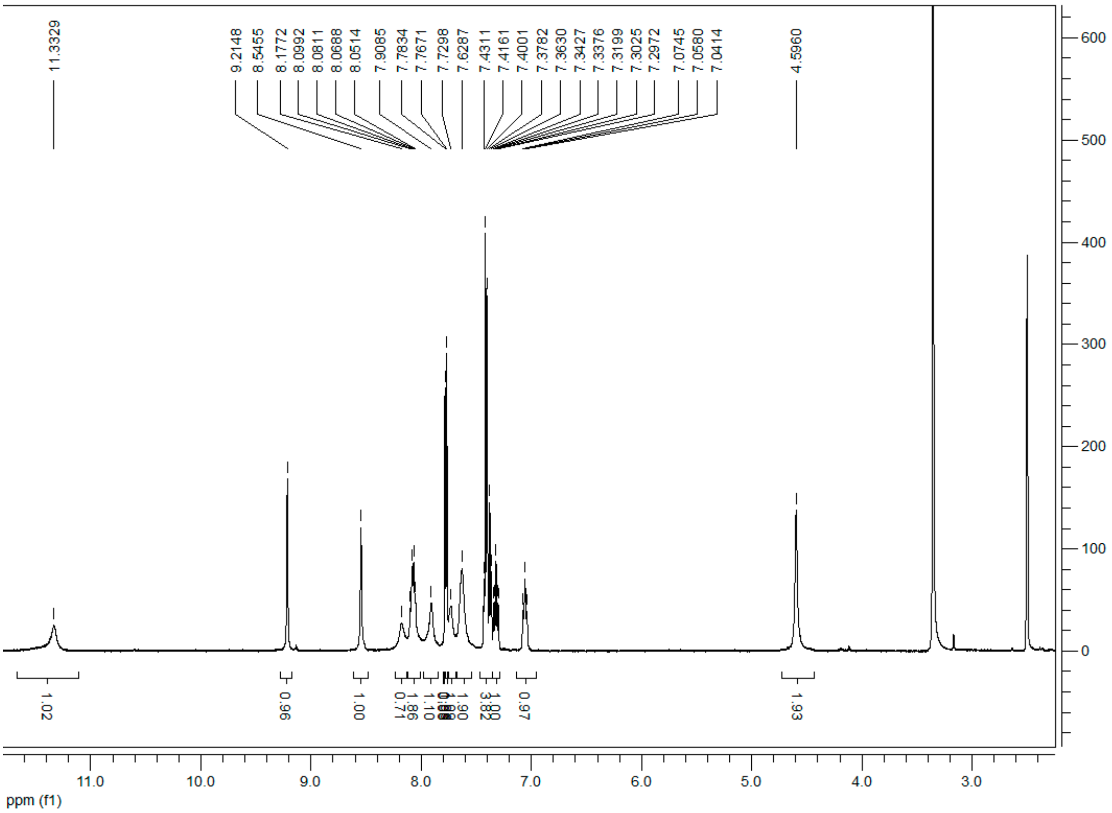

Figure S32. ^{1}H-NMR of compound **4c** (500 MHz, DMSO-d_6).

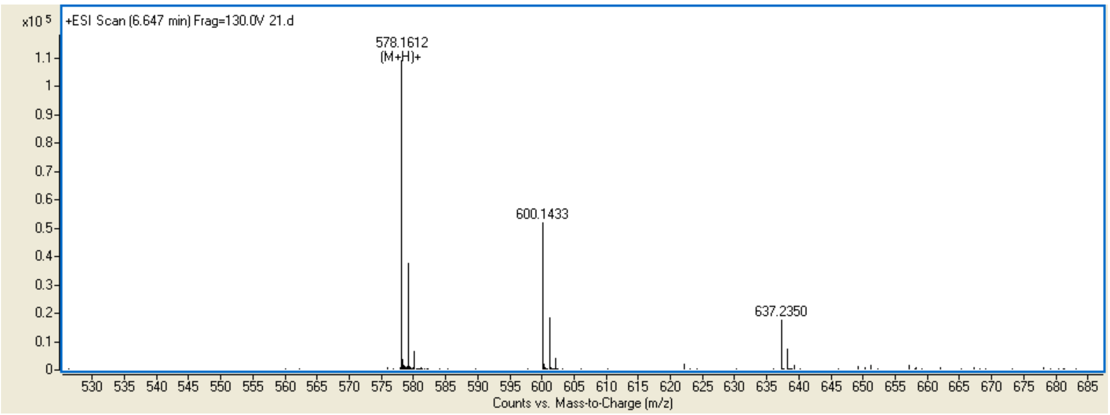

Figure S33. HR-ESI-MS of compound **4c**.

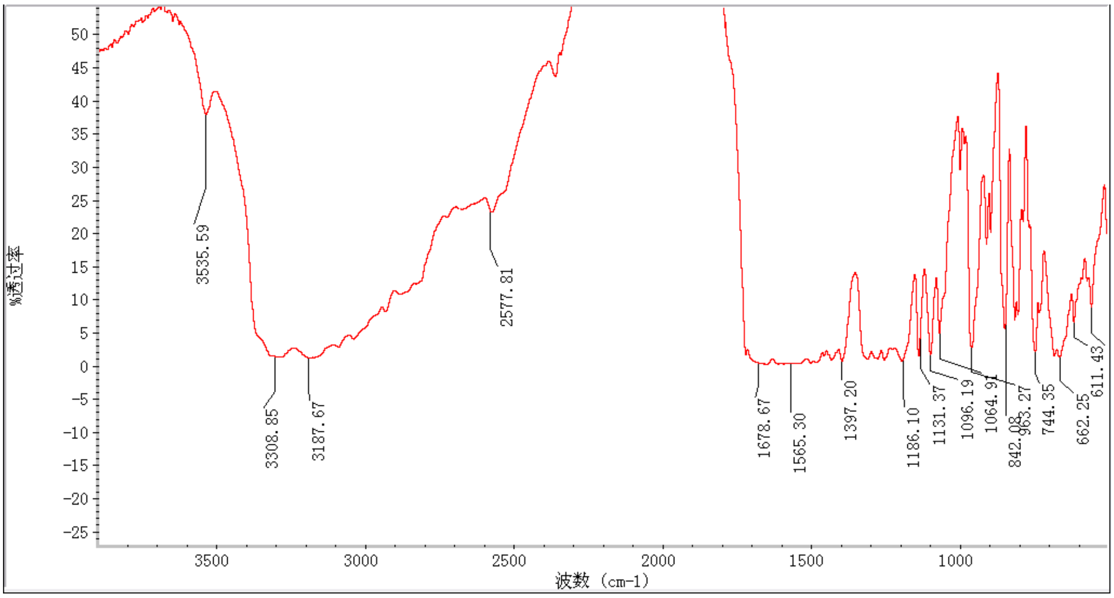

Figure S34. IR of compound **4c**.

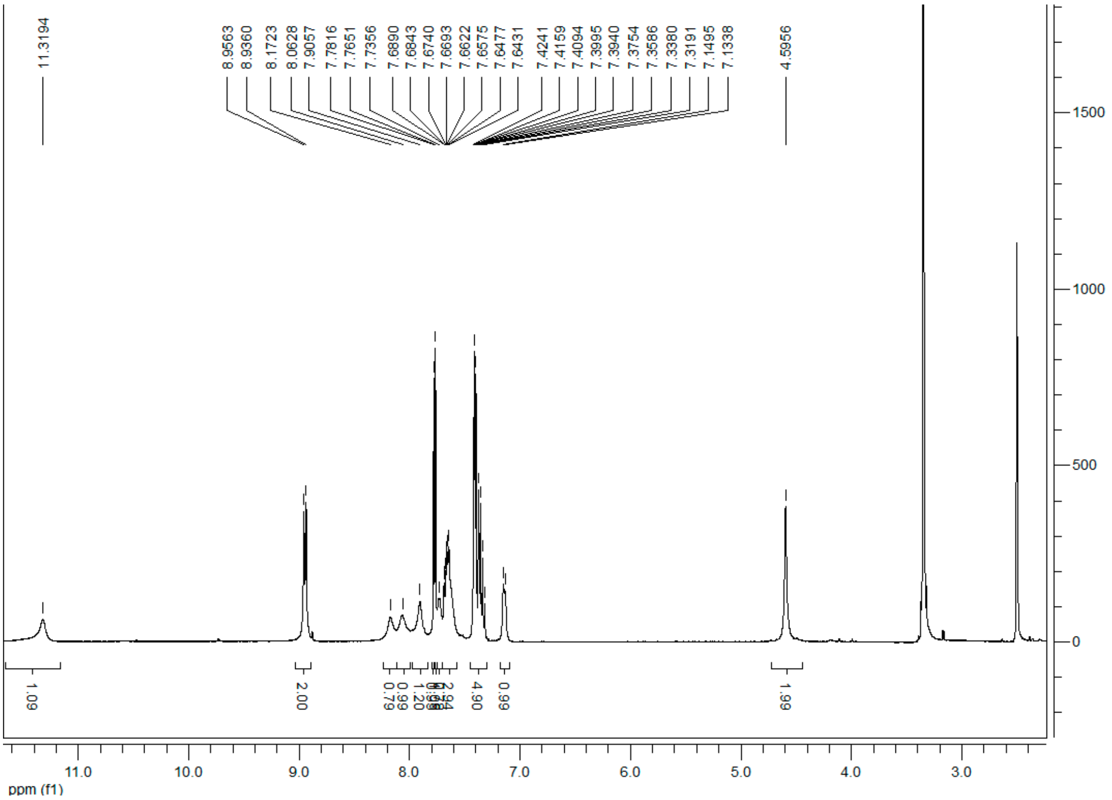

Figure S35. ^{1}H-NMR of compound **4d** (500 MHz, DMSO-d_6).

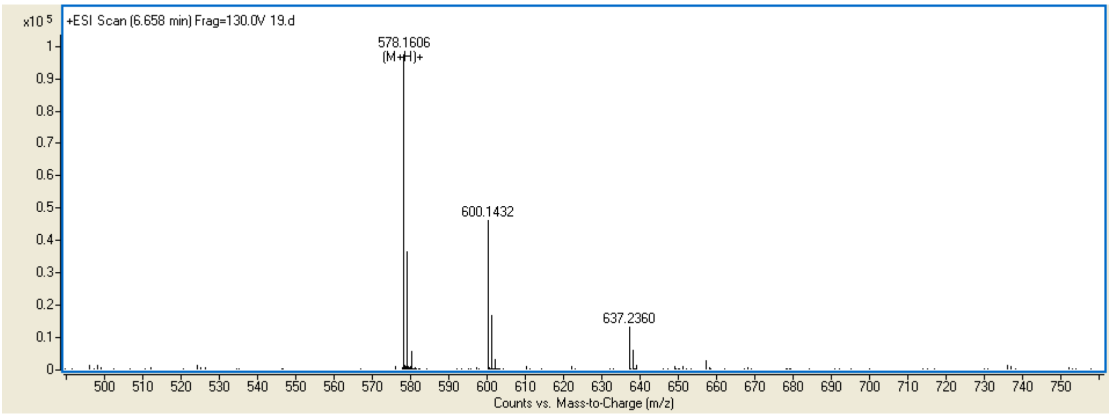

Figure S36. HR-ESI-MS of compound **4d**.

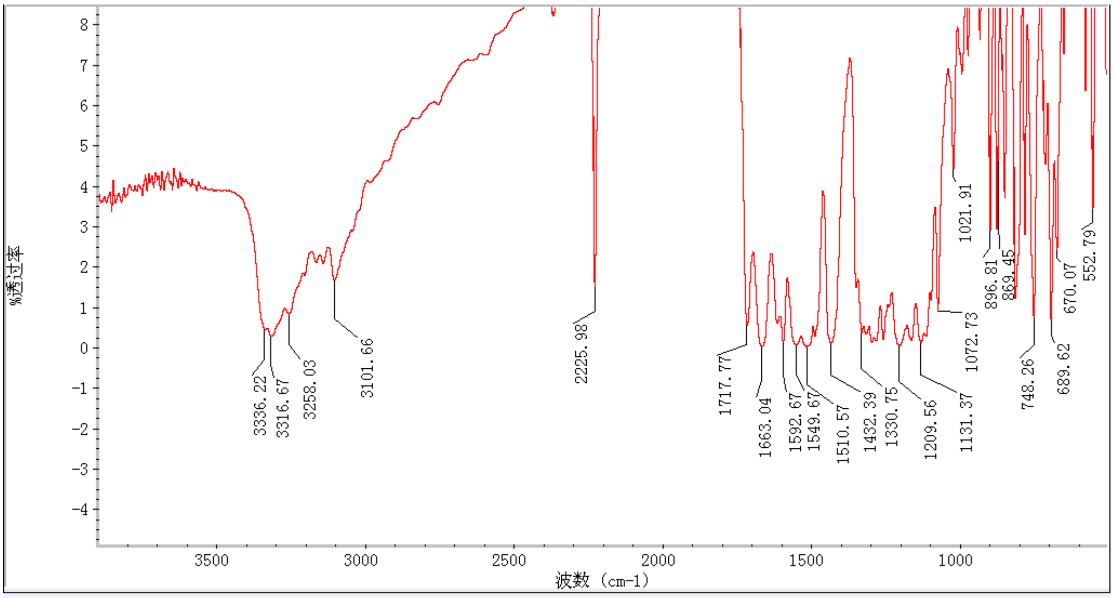

Figure S37. IR of compound **4d**.

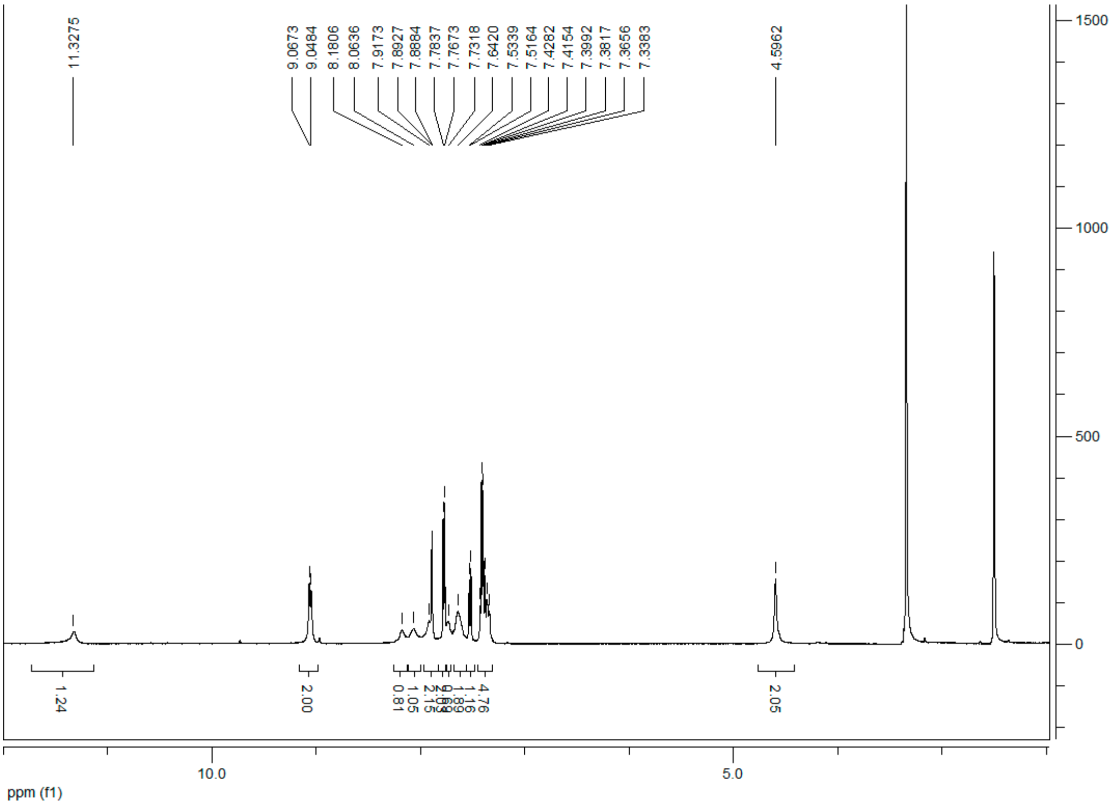

Figure S38. ^{1}H-NMR of compound **4e** (500 MHz, DMSO-d_6).

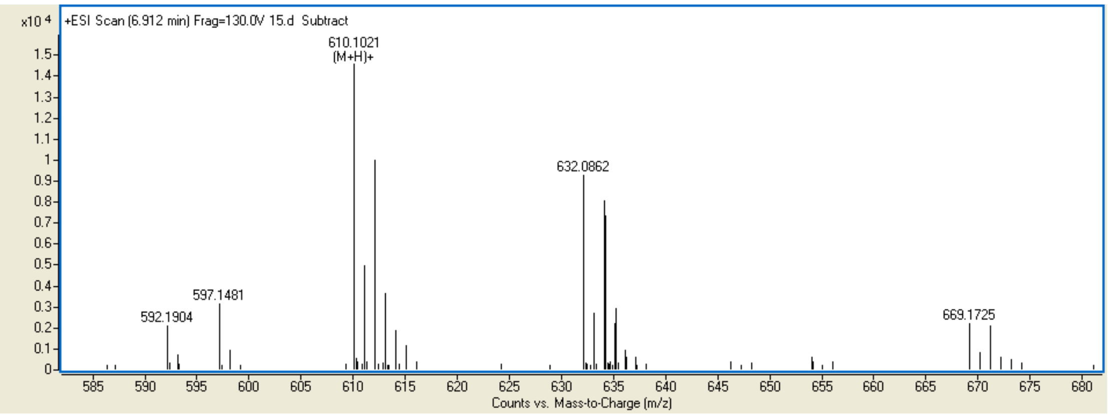

Figure S39. HR-ESI-MS of compound **4e**.

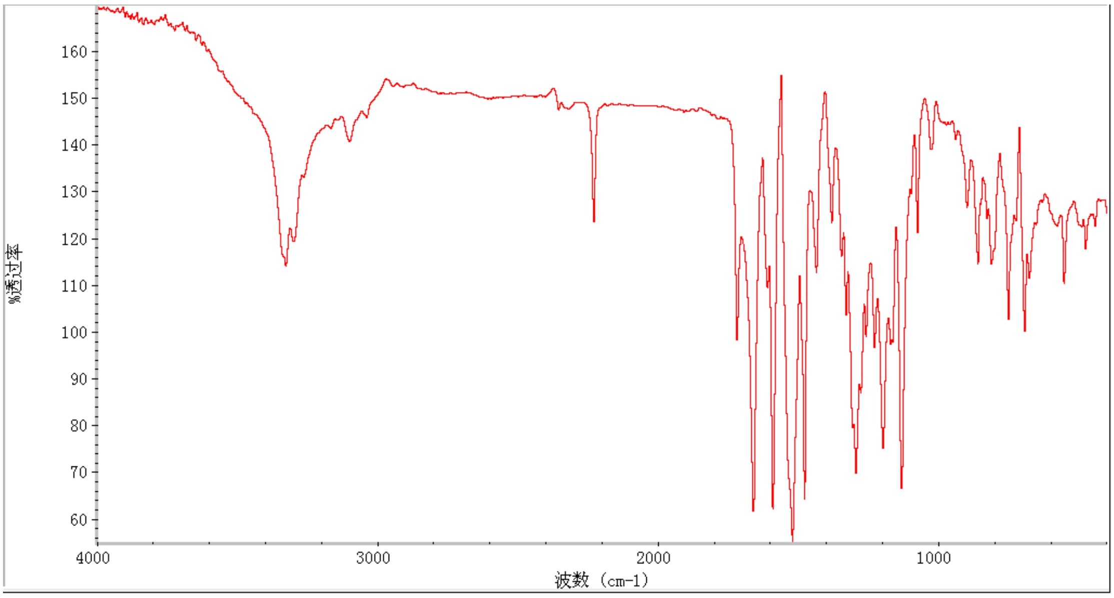

Figure S40. IR of compound **4e**.

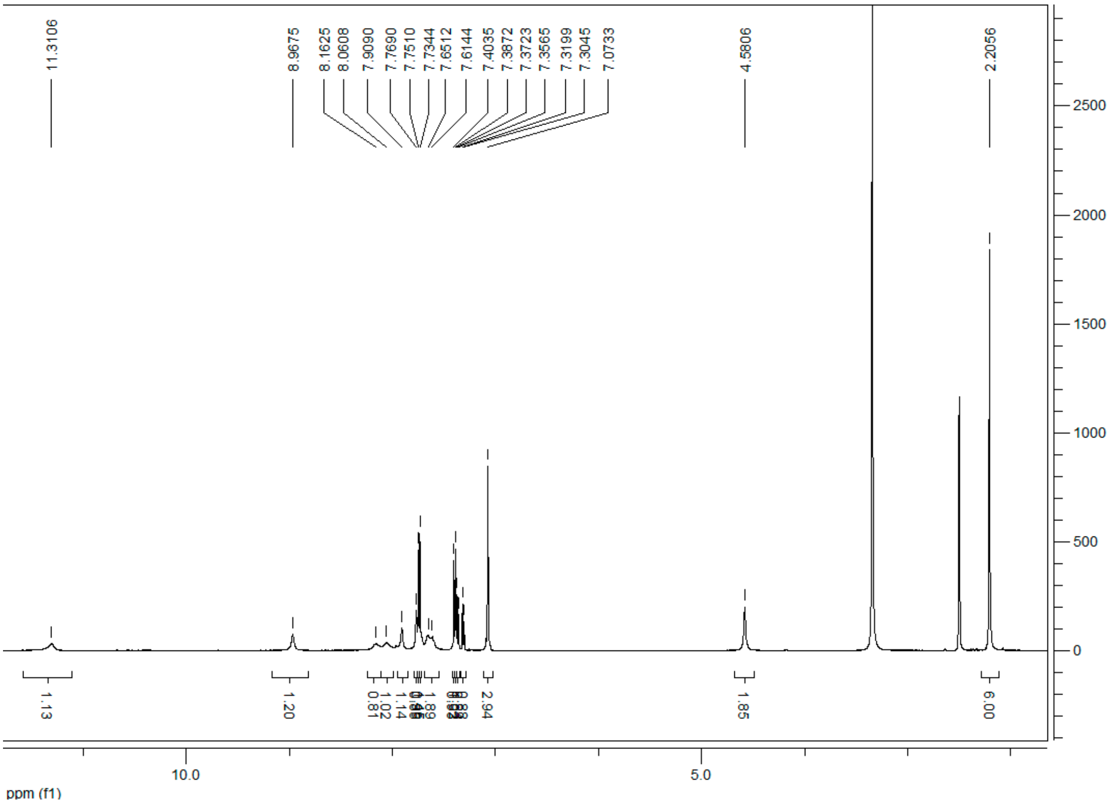

Figure S41. ^{1}H-NMR of compound **4f** (500 MHz, DMSO-d_6).

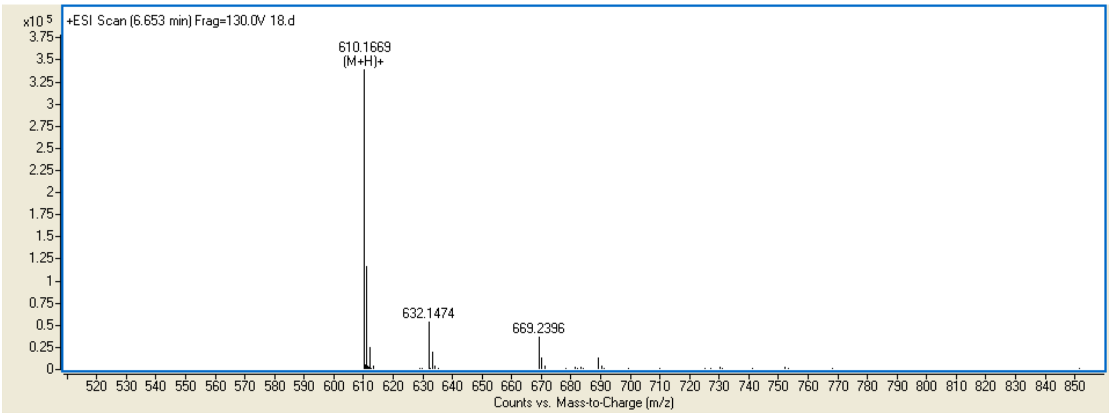

Figure S42. HR-ESI-MS of compound **4g**.

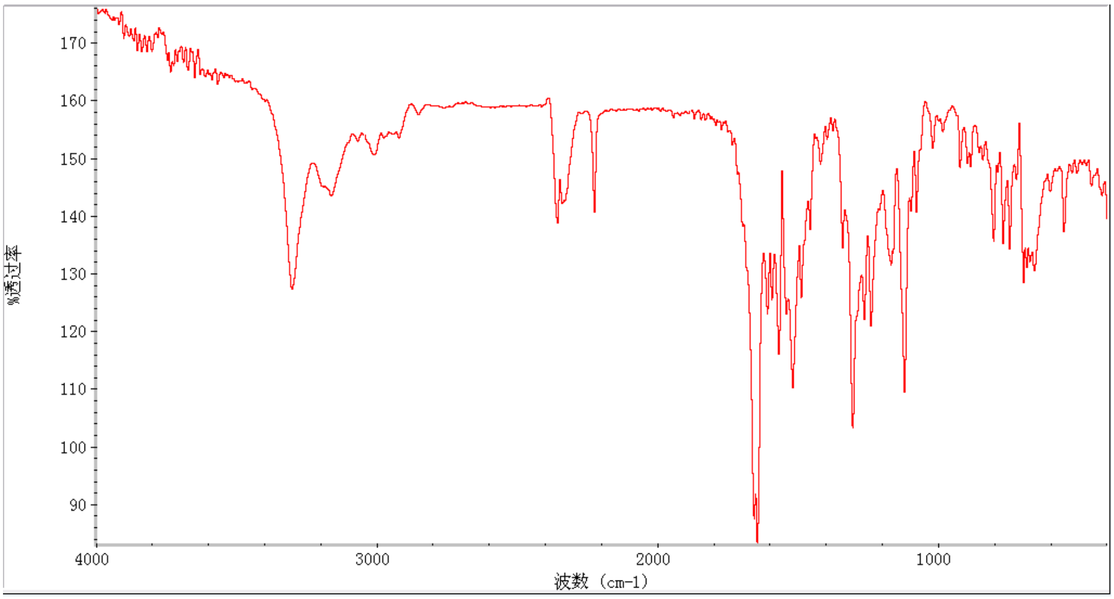

Figure S43. IR of compound **4f**.

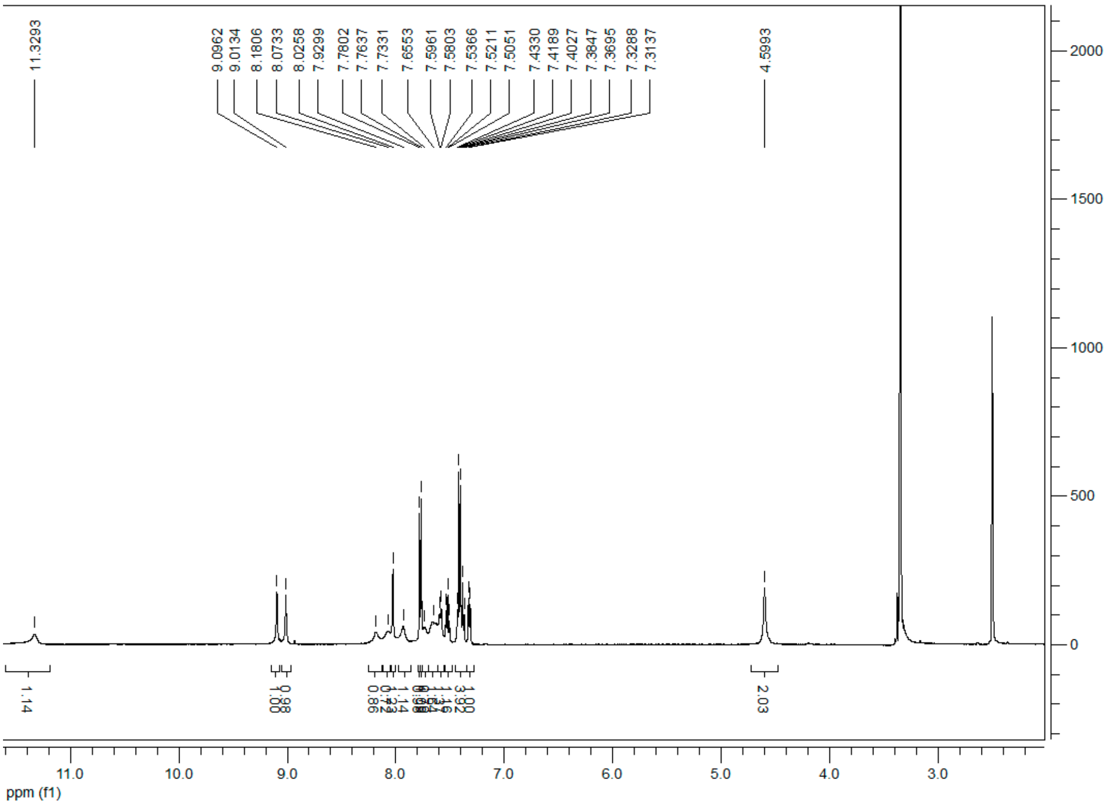

Figure S44. ^{1}H-NMR of compound **4g** (500 MHz, DMSO-d_6).

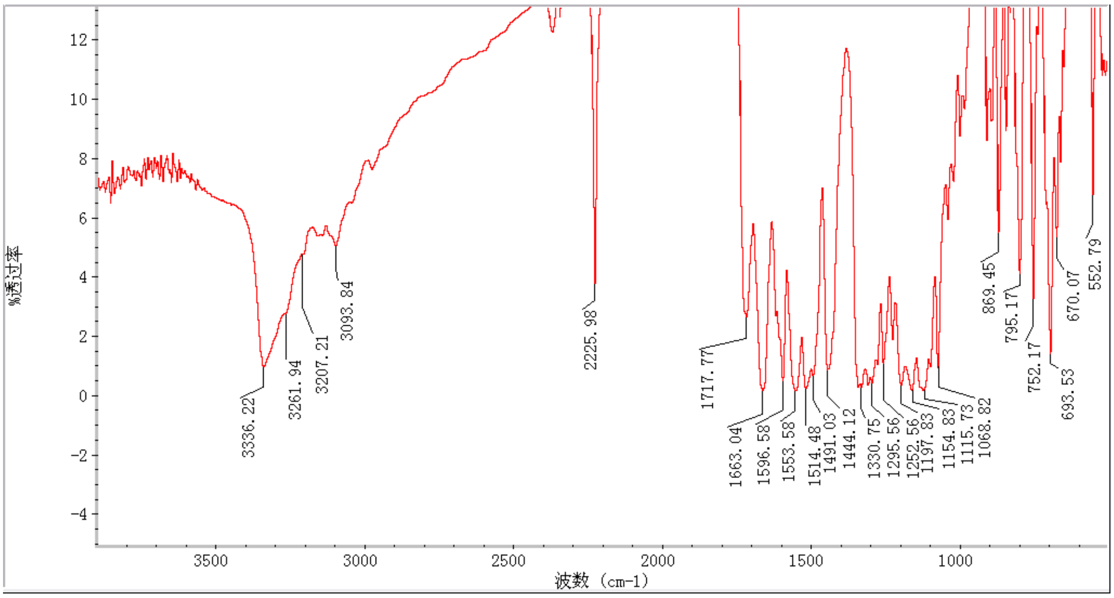

Figure S45. IR of compound **4g**.

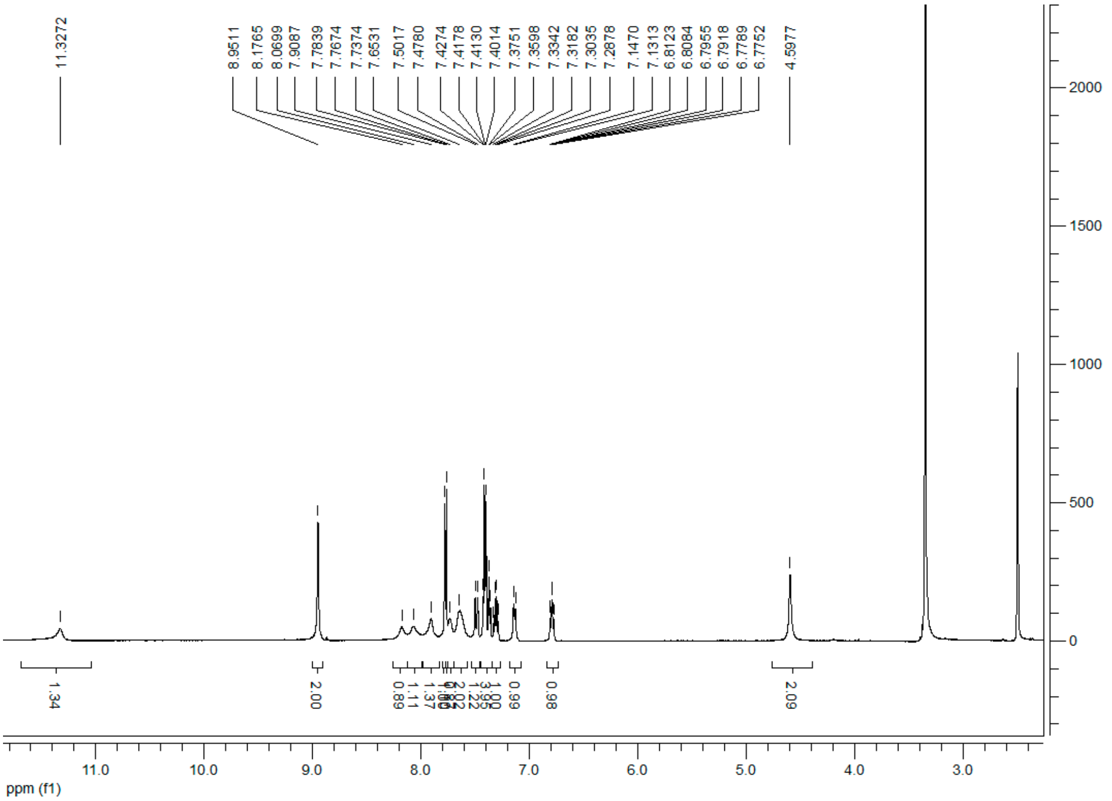

Figure S46. ^{1}H-NMR of compound **4h** (500 MHz, DMSO-d_6).

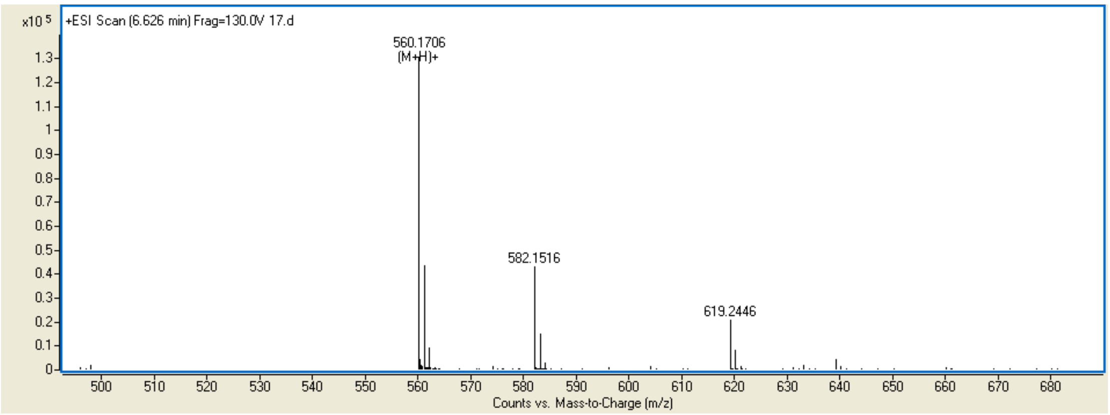

Figure S47. HR-ESI-MS of compound **4h**.

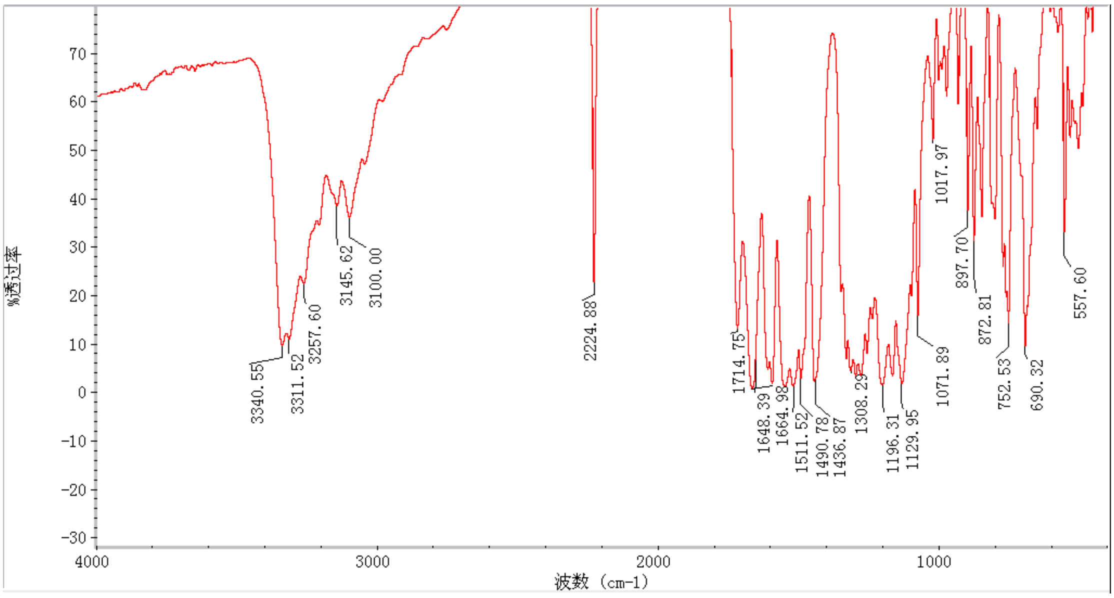

Figure S48. IR of compound **4h** (500 MHz, DMSO-d_6).

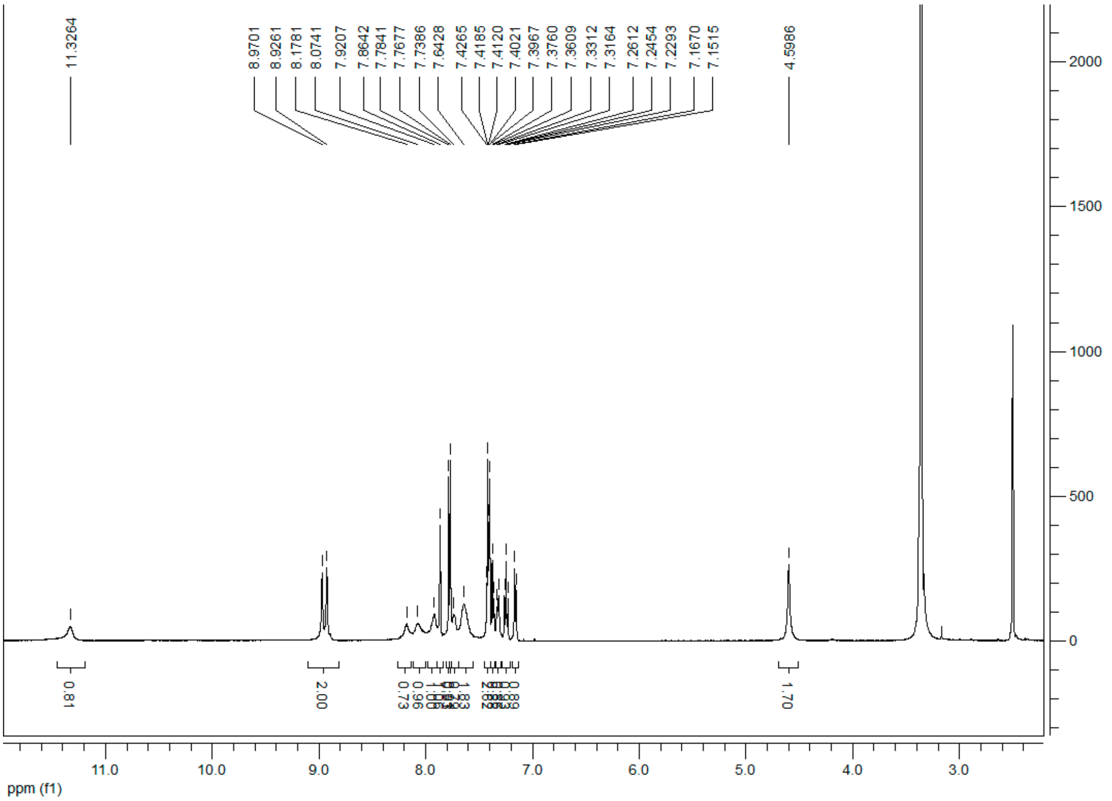

Figure S49. ^{1}H-NMR of compound **4i** (500 MHz, DMSO-d_6).

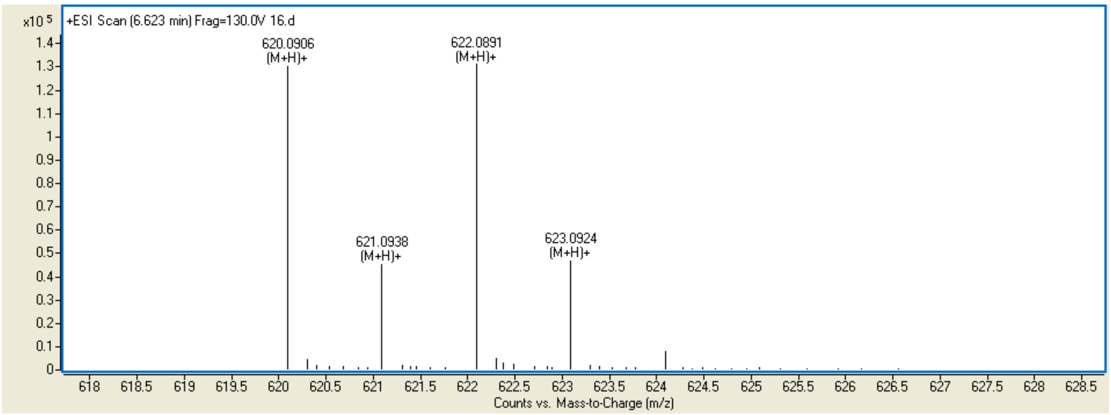

Figure S50. HR-ESI-MS of compound **4i**.

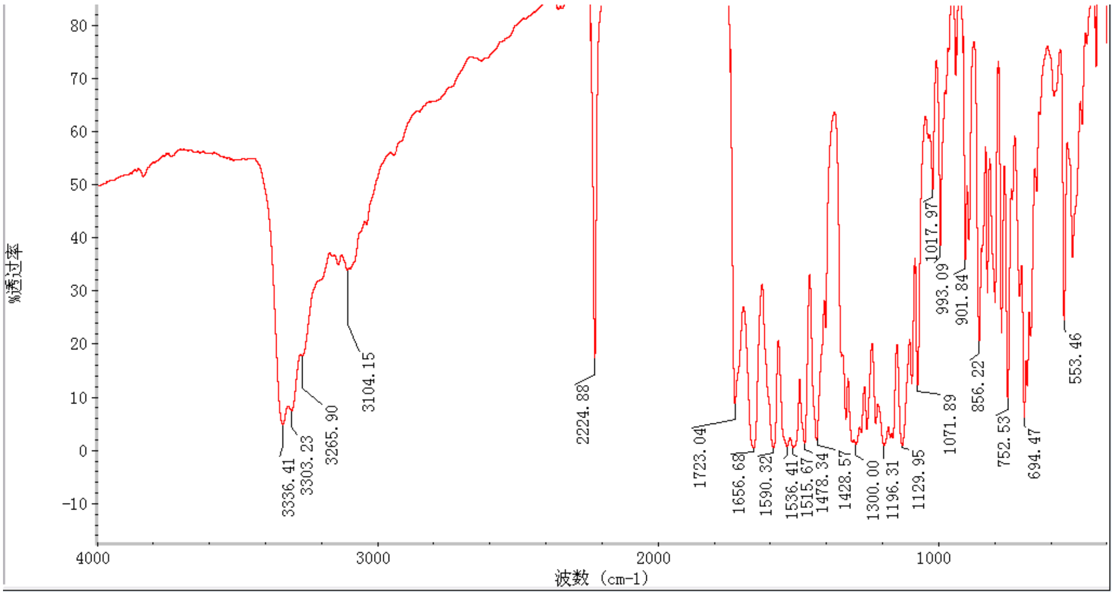

Figure S51. IR of compound **4i**.
